# Analysis of temporal changes in HIV-1 CRF01_AE *gag* genetic variability and CD8 T-cell epitope evolution

**DOI:** 10.1371/journal.pone.0267130

**Published:** 2022-05-10

**Authors:** Wardah Rafaqat, Uroosa Tariq, Nida Farooqui, Maheen Zaidi, Aanish Raees, Maaz Zuberi, Amna Batool, Syed Hani Abidi

**Affiliations:** 1 Medical College, Aga Khan University, Karachi, Pakistan; 2 Department of Biological and Biomedical Sciences, Aga Khan University, Karachi, Pakistan; 3 Cincinnati Children’s Hospital Medical Center, Cincinnati, Ohio, United States of America; Shanghai Public Health Clinical Center, CHINA

## Abstract

Currently, little is known about the time-dependent evolution of human immunodeficiency virus-1 (HIV-1) circulating recombinant forms (CRF) 01_AE, a dominant recombinant form associated with HIV-1 epidemics worldwide. Since *gag* is a highly immunodominant HIV-1 protein, we performed a comparative analysis of the CRF01_AE *gag* protein’s time-dependent changes and evolution. A total of 3105 HIV-1 CRF01_AE *gag* sequences representing 17 countries from the timeline 1990–2017 were obtained. The sequences’ phylogenetic relationship and epidemic dynamics were analyzed through a Maximum Likelihood tree and Bayesian Skyline plot, respectively. Genomic variability was measured through Shannon entropy and time-dependent immunoevolution was analyzed using changes in proteasomal degradation pattern, cytotoxic T lymphocytes (CTL) epitopes, and Human leukocyte antigens (HLA) restriction profile. The most recent common ancestor of the HIV CRF01_AE epidemic was estimated to be 1974±1. A period of exponential growth in effective population size began in 1982, fluctuated, and then stabilized in 1999. Genetic variability (entropy) consistently increased, however, epitope variability remained comparable; the highest number of novel CTL epitopes were present in 1995–1999, which were lost over time. The spread of the HIV-1 CRF01_AE epidemic is predominant in countries within Asia. Population immunogenetic pressures in the region may have contributed to the initial changes and following adaptation/stabilization of epitope diversity within *gag* sequences.

## Introduction

HIV remains a prevalent global health issue worldwide [1]. HIV-1 is divided into 3 major groups; M, N, and O, where group M is further diversified into subtypes and several circulating recombinant forms [2]. One such recombinant form is HIV-1 CRF01_AE.

CRF01_AE was previously believed to have originated in Central Africa and spread to Thailand in the late 1970s [3]. From Thailand, the transmission may have occurred in Vietnam and China through key population groups, including female sex workers and people who inject drugs [4]. However, recent phylogenetic analysis suggests that instead of originating only from Central Africa, there may have been multiple CRF01_AE founder viruses present in China which then spread within high-risk groups in the country [5].

Currently, HIV-1 CRF01_AE comprises 5.9% of total world sequences (www.hiv.lanl.gov). It is found in regions including North America, Western Europe, Eastern Europe, and Central Asia, South and Southeast Asia, East Asia, Oceania, and Central Africa with the highest prevalence in South and Southeast Asia [6]. Within Southeast Asia, CRF01_AE is responsible for the majority of HIV infections in countries including China, Thailand, Cambodia, Vietnam, Philippines, Malaysia, Singapore, and Indonesia (www.hiv.lanl.gov). The *gag* gene of HIV-1 CRF01_AE contributes to the structural maturation of the virus by producing higher-ordered structures for the correct assembly, budding, and maturation of new infectious particles [7, 8]. It has a multimerized structure which makes it suitable as a target to be used in an antiviral vaccine [9]. It is a highly immunogenic protein, containing some dominant epitopes that are frequently targeted by CD8+ T-cells [10, 11]. Mutations in *gag* protein can interfere with the processing of viral antigen, thus disrupting the generation of viable antigenic epitopes. The patient’s human leukocyte antigen (HLA) alleles represent a major host-driven selective pressure that steers the amplification of specific epitope mutations [10, 12, 13].

Therefore, understanding the origin and evolutionary changes of *gag* HIV-1 CRF01_AE is necessary to understand HIV-1 CRF01_AE genetic diversity, resistance patterns, molecular epidemiology, and immune evolution [14]. This study aimed to analyze the pan-epidemic, time-dependent genetic variation, and epitope evolution analysis of the HIV-1 CRF01_AE *gag* gene.

## Materials and methods

### HIV-1 subtype CRF01_AE *gag* sequences

A total of 3624 full-length HIV-1 CRF01_AE *gag* sequences were retrieved from the HIV Los Alamos Database (http://www.hiv.lanl.gov/), and after removal of duplicate sequences, a total of 3105 sequences were used in the study. These sequences were deposited from the following 17 countries: Afghanistan, Cameroon, Central African Republic, China, Cyprus, Hong Kong, Indonesia, Ireland, Japan, Myanmar, Philippines, Sweden, Switzerland, Thailand, United Kingdom, United States, and Vietnam (S1 Table).

### Collection and year-wise grouping of HIV-1 CRF01_AE sequences

From the total of 3105 HIV-1 CRF01_AE full-length *gag* sequences, the oldest sequence was from 1990 from Thailand and the Central African Republic, while the most recent sequences were from 2017 from the Philippines. *Gag* sequences were divided into five groups, namely 1990–1994, 1995–1999, 2000–2004, 2005–2009, 2010–2014, and 2015–2017 comprising 28, 42, 445, 1517, 986, and 87 sequences, respectively. All groups contained data from 5 years except for the 2015–2017 group which covered 3 years (inclusive of years 2015 and 2017; S1 Table)). All sequences were aligned using MEGA7 software, using the ClustalW algorithm. Unless stated otherwise, for all analyses performed, the same grouping of sequences was maintained.

### Phylogenetic analysis and determination of effective population size and time to the most recent common ancestor (tMRCA)

A Maximum Likelihood (ML) tree was generated with 3105 *gag* sequences using PhyML software [15], using the Generalized Time Reversible (GTR) model of nucleotide substitution and approximate likelihood ratio test (aLRT) and the Shimodaira–Hasegawa (SH)-aLRT measure of branch support. The phylogenetic tree was used to identify clusters with strong (>0.8) aLRT node values.

A Bayesian Markov Chain Monte Carlo (MCMC) inference was applied to estimate the effective population size and tMRCA of HIV-1 CRF01_AE, using BEAST v1.7.4 software. For this analysis, a total of 286 *gag* sequences from different countries from the years 1990 to 2017 were used. Sequences were selected using the approach described by Novitsky, et al., where a limit was set to 5–6 sequences per country and year groups [16]. If 5 or fewer sequences were represented in a country, all of them were included in the analysis. Bayesian analysis was performed using the following parameters: lognormal relaxed molecular clock model, general time-reversible (GTR) nucleotide substitution model, estimated base frequencies, and gamma distribution model for heterogeneity among nucleotide sites. The analysis was performed using demographic models of constant population size and a Bayesian skyline plot [17]. The MCMC chain length was set at 2×10^8^, which gave an effective sample size (ESS) of >200. MCMC sample analysis and Bayesian skyline plot construction were performed using Tracer v1.7.4 [18].

### Analysis of time-dependent genomic variability and CD8 T cell epitope evolution

To evaluate time-dependent changes in HIV-1 CRF01_AE genomic variability, Shannon entropy analyses were performed. Shannon entropy is a measure of the probability of acquiring mutations, including epitope-related mutations, in a given set of genomic sequences [19]. The Shannon entropy of HIV-1 CRF01_AE *gag* sequences from each year group was calculated using an online tool available at the Los Alamos National Laboratory (LANL) HIV Sequence Database: http://www.hiv.lanl.gov/content/sequence/ENTROPY/entropy_one.html. Using GraphPad software, the mean entropy value for each year group was calculated, while the statistical significance of the difference between the mean of each year group was calculated using one-way ANOVA with Tukey’s multiple comparison test.

Proteasomal degradation sites were predicted in the HIV-1 CRF01_AE *gag* sequences using NetChop 3.1 software [20]. A predictive score on a scale of 0–1.0 was used to measure the probability of the existence of a proteasomal site, where sites with a cut-off score of 0.5 or higher were considered proteasomal degradation sites.

For epitope analysis, consensus *gag* gene sequences for each year group were created using the Consensus Maker tool, and consensus sequences were translated into amino acid sequences using the Expasy translate tool. These protein sequences were then used to predict CD8+ T cell epitopes using CTLPred software [21], where an Artificial Neural Network (ANN) cutoff score of 0.51, and SVM cutoff score of 0.36 was used. Peptides with a score higher than the given values were identified as CTL epitopes. The software allowed the identification of epitopes that may be crucial in vaccine design. Additionally, MHC restriction for *gag* epitopes was predicted using nHLAPred software [22], where the ANN and Quantitative Matrix (QM) filters were set at a default value of 0.5; thus, any peptide achieving a score above the threshold value was recognized and was, therefore, a potential MHC binder. This software was also used to predict restricting Human Leukocyte Antigen (HLA-I) for peptides/epitopes. Using this approach, for a given peptide/epitope, all possible restricting class I HLAs were identified. To evaluate time-dependent epitope evolution, genomic variability, mutations in the epitope sequence, changes in CTL epitopes, and changes in HLA restriction sites over the period were observed.

The epitope analysis was also confirmed using HIV-1 CRF01_AE *env* sequences. For this analysis, a total of 5265 *env* sequences (S2 Table) were retrieved from the HIV Los Alamos Database (http://www.hiv.lanl.gov/) and grouped as per the strategy described above except for the last group (2015–2018) which contained one sequence for 2018. The epitope analysis was performed as described above for the gag protein.

## Results

### Analysis of the phylogenetic relationships, origin, and global epidemic dynamics of HIV-1 CRF01_AE

**Fig 1:**
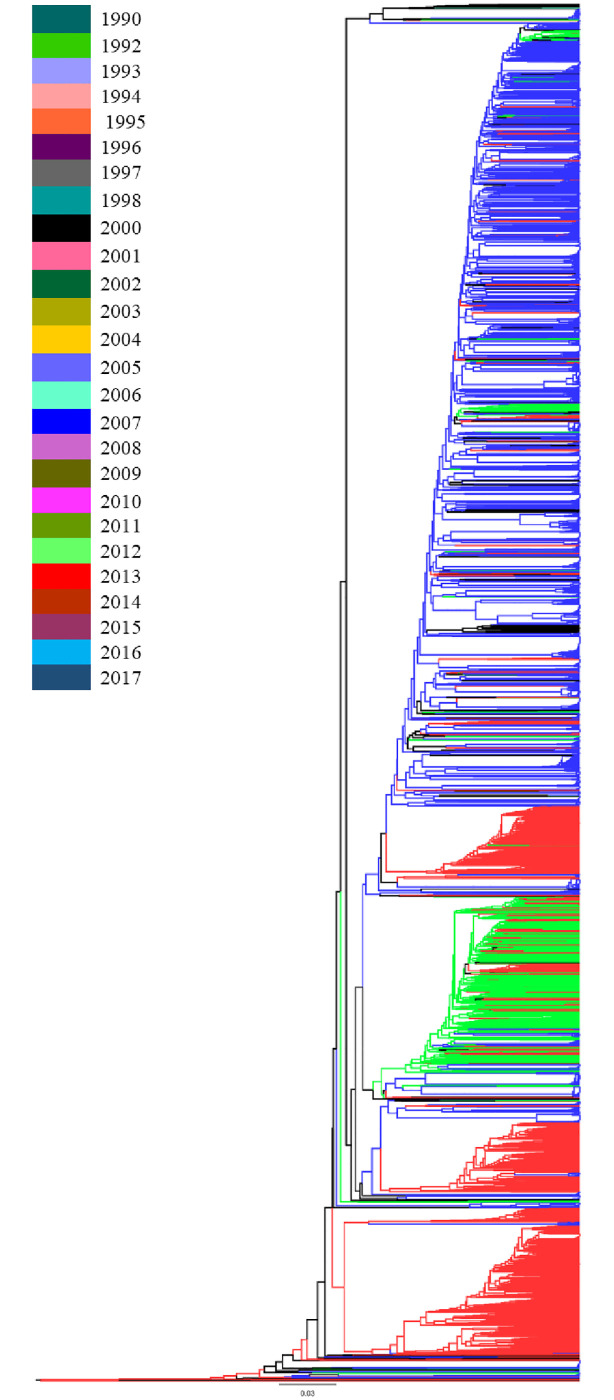
Maximum likelihood (ML) tree of HIV-1 CRF01_AE *gag* gene sequences. ML tree was used to infer the phylogenetic relationship among the 3105 HIV-1 CRF01_AE *gag* sequences submitted to the LANL HIV Sequence Database, representing the years 1990 to 2017. The tree was color-coded based on the years groups. The color key for the tree is given within the figure.

**Fig 2:**
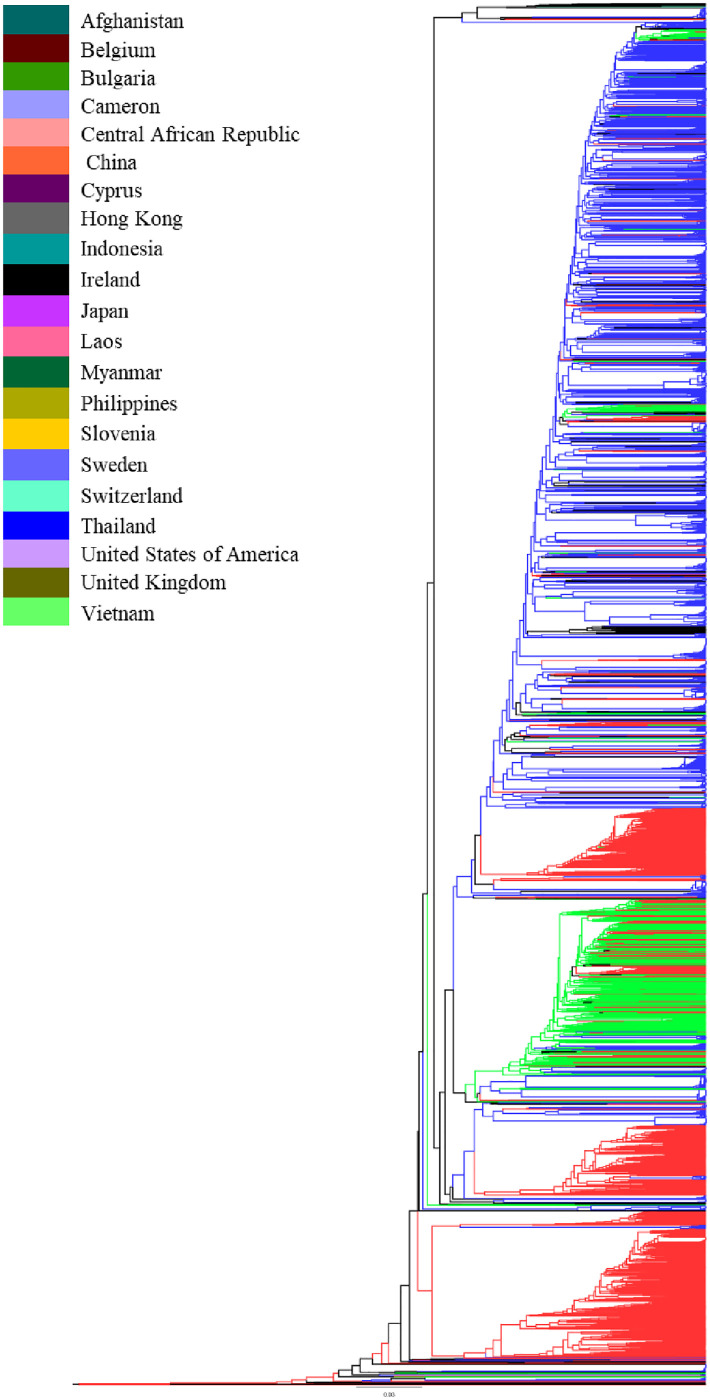
Maximum likelihood (ML) tree of HIV-1 CRF01_AE *gag* gene sequences. ML tree was used to infer the phylogenetic relationship among the 3105 HIV-1 CRF01_AE *gag* sequences submitted to the LANL HIV Sequence Database, representing the years 1990 to 2017. The tree was color-coded based on the countries. The color key for the tree is given within the figure.

Phylogenetic relationships among the HIV-1 CRF01_AE *gag* sequences revealed phylogenetic clustering between the sequences from the years 2013, 2010, and 2005. Sequences from the other years were scattered throughout the phylogenetic tree, without any apparent relationship between branching topology and time of sampling (Fig 1). Analysis of the phylogenetic tree according to country showed cluster formation between sequences from China, Thailand, and Vietnam, while sequences from the remaining countries were scattered throughout the phylogenetic tree (Fig 2).

**Fig 3:**
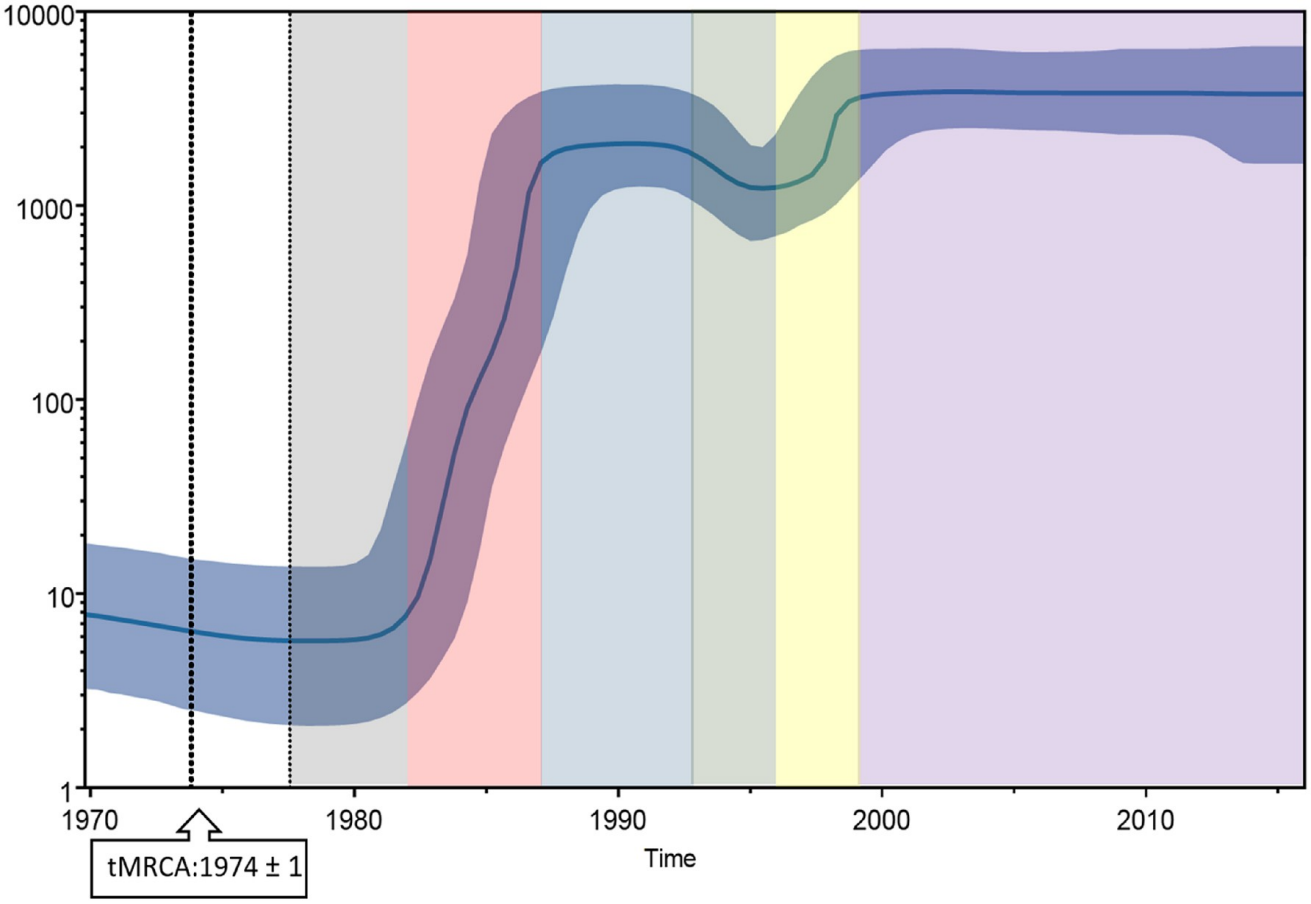
HIV-1 CRF01_AE *gag* gene effective population size and time to the most recent common ancestor. Bayesian Skyline plot, based on a ‘relaxed clock’ coalescent framework analysis, was constructed using 286 sequences (representing all years and countries). The X-axis represents time in years, while Y-axis shows an effective population size. The thick black line represents the median, while the blue band represents 95% highest posterior density (HPD) intervals. The tMRCA of the HIV-1 subtype AE *gag* gene is indicated by a black dotted line and a black box. Grey, red, blue, green, yellow, and purple shaded areas represent the period of the plateau phase, increase in viral effective population size, plateau phase, decline, increase, and plateau, respectively.

To explore the relationship between HIV-1 CRF01_AE *gag* gene divergence and epidemic dynamics, Bayesian Skyline analysis was performed. The estimated time to the most recent common ancestor (tMRCA) for CRF01_AE was found to be around 1974±1 (Fig 3, black dotted line). Compared to the tMRCA, the Bayesian Skyline plot for *gag* identified at least an initial 100-fold growth in viral effective population size (correlating with an effective number of infections and/or transmission opportunities [23]) between 1982 to 1988 (Fig 2, red region). There was a decline in effective population size between 1992–1996, where an approximately 3-fold decrease was observed (Fig 3, green region). This was followed by a 3-fold rise in effective population size between 1995–1999 (Fig 3, yellow region) after which, the effective population size attained plateaued (Fig 3, purple region).

### Time-dependent genetic evolution of HIV-1 CRF01_AE *gag* sequences

**Fig 4:**
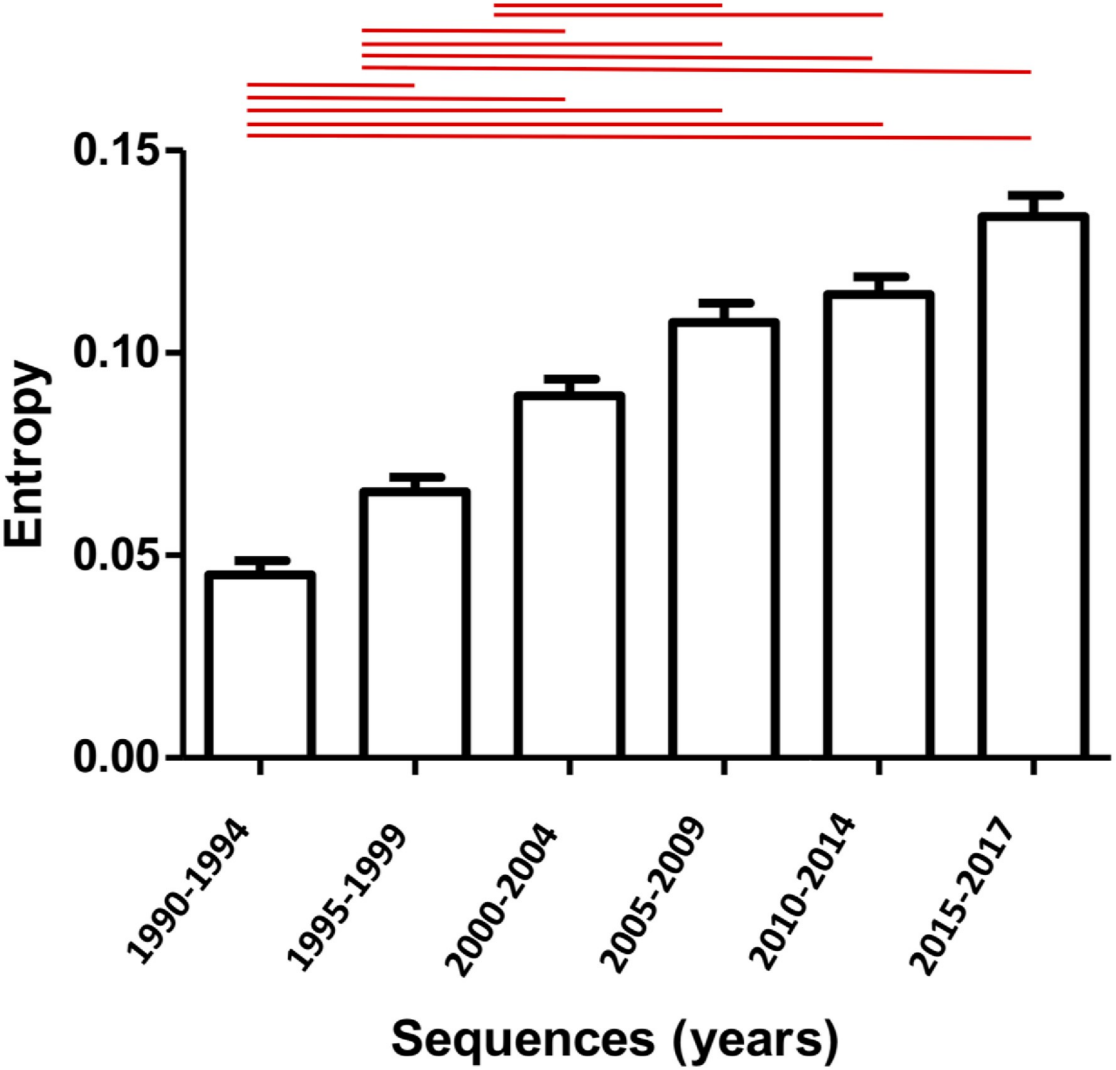
Time-dependent changes in HIV-1 CRF01_AE *gag* genomic variability. The mean entropy score for each year group was calculated and plotted using GraphPad software. The Redline over bars represents a statistically significant difference between the groups (p<0.05). Error bars represent the standard error of the mean.

To analyze HIV-1 CRF01_AE *gag* variability over the study period, we performed the Shannon entropy analysis. The *gag* sequences were grouped into the following year sets: 1990–1994, 1995–1999, 2000–2004, and 2005–2009, 2010–2014, and 2015–2017. Shannon Entropy analysis showed a consistent increase in entropy over the years, with the highest value observed for the 2015–2017 year group (Fig 4). Moreover, this difference in mean entropy value of the first three year groups with all other year groups was significant (p<0.05), while the difference in mean entropy between the 2000–2005 and 2015–2017 and 2005–2010 and 2010–2015 was statistically insignificant (Fig 4).

### Time-dependent HIV-1 CRF01_AE *gag* epitope evolution

**Fig 5:**
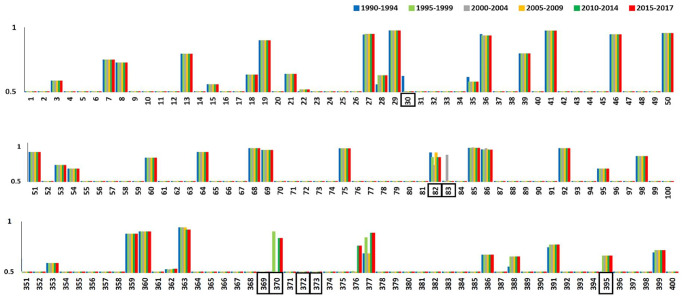
Proteasomal degradation sites in HIV-1 CRF01_AE *gag* protein. Proteasomal degradation sites *gag* protein sequences from 1990–1994 (blue), 1995–1999 (light green), 2000–2004 (grey), 2005–2009 (yellow), 2010–2014 (dark green), and 2015–2017 (red) year groups were predicted using NetChop software. Below the sequence, the numbers indicating the position of each amino acid with reference to the HXB2 reference strain are shown. Only proteasomal degradation sites with a cut-off value >0.5 are shown. The black box indicates the position at which the mutation occurred.

The proteasome machinery degrades proteins into peptides of varying lengths, which are then sequentially processed and displayed by the HLA-I molecules to T lymphocytes. Comparative analysis of the sequences from both groups showed that most of the proteasomal sites were common between different year groups. There were, however, a few sites, namely 30, 82, 83, 369, 370, 372, 373, and 395 (with reference to HBX2 *gag* protein) where mutations were present, while differences in proteasomal degradation scores were noted at sites 30, 82, 83, and 370 (with reference to HBX2 *gag* protein; Fig 5). These mutations resulted in changes in epitope sequences observed between the different year groups (Table 1). Additionally, mutation at position 370 resulted in the emergence of unique epitopes in certain year groups, for example, epitopes AQHANIMMQ and SQAQHANIM, which were observed only in 1990–1994, 2000–2004, and 2005–2009 year groups (Table 1).

**Table 1:** HIV-1 CRF01_AE *gag* CTL mutated and novel epitopes. The epitopes are divided into three categories: Novel epitopes (epitopes unique to one year group), intermittently recurring epitopes and mutated epitopes. Mutation(s) in epitope are underlined, while ‘-‘ in the table represents the absence of epitope in a particular year group.

	1990–1994	1995–1999	2000–2004	2005–2009	2010–2014	2015–2017
**Mutated epitopes**	GKKKYKMKH	GGKKKYRMK	GGKKKYRMK	GGKKKYRMK	GGKKKYRMK	GGKKKYRMK
	RPEPTAPPA	RTEPTAPPA	RPEPTAPPA	RPEPTAPPA	RPEPTAPPA	RPEPTAPPA
	KYKMKHLVW	KYRMKHLVW	KYRMKHLVW	KYRMKHLVW	KYRMKHLVW	KYRMKHLVW
	YKMKHLVWA	YRMKHLVWA	YRMKHLVWA	YRMKHLVWA	YRMKHLVWA	YRMKHLVWA
	ELKSLFNTV	ELKSLFNTI	ELKSLFNTV	ELKSLFNTV	ELKSLFNTI	ELKSLFNTI
	SLFNTVATL	SLFNTIATL	SLFNTVVTL	SLFNTVATL	SLFNTIATL	SLFNTIATL
	DKIEEVQNK	DKIEEVQNK	DKIEEVQNK	DKIEEVQNK	DKIEEVQKK	DKIEEVQKK
	IEEVQNKSQ	IEEVQNKSQ	IEEVQNKSQ	IEEVQNKSQ	IEEVQKKSQ	IEEVQKKSQ
	MTNNPPIPV	MTNNPPIPV	MTNNPPIPV	MTNNPPIPV	MTSNPPIPV	MTSNPPIPV
	RVLAEAMSQ	RVLAEAMSH	RVLAEAMSQ	RVLAEAMSQ	RVLAEAMSQ	RVLAEAMSQ
	RIKCFNCGR	RIKCFNCGK	RIKCFNCGK	RIKCFNCGK	RIKCFNCGK	RIKCFNCGK
	QAQHANIMM	HVQHANIMM	QAQHANIMM	QAQHANIMM	QVQQTNIMM	QVQQTNIMM
	KKYKMKHLV	KKKYRMKHL	KKKYRMKHL	KKKYRMKHL	KKKYRMKHL	KKKYRMKHL
	-	VLAEAMSHV	-	-	VLAEAMSQV	VLAEAMSQV
**Intermittently recurring epitopes**	-	NWGMGEEIT	-	-	NWGMGEEIT	NWGMGEEIT
	AQHANIMMQ	-	AQHANIMMQ	AQHANIMMQ	-	-
	SQAQHANIM	-	SQAQHANIM	SQAQHANIM	-	-
	-	-	-	-	GGPSHKARV	GGPSHKARV
	KIEEVQNKS	KIEEVQNKS	KIEEVQNKS	KIEEVQNKS	-	-
	DIAGTTSTL	DIAGTTSTL	DIAGTTSTL	DIAGTTSTL	-	-
	-	EELKSLFNT	-	-	EELKSLFNT	EELKSLFNT
	-	KSLFNTIAT	-	-	KSLFNTIAT	KSLFNTIAT
	ATLWCVHQR	ATLWCVHQR	-	ATLWCVHQR	ATLWCVHQR	ATLWCVHQR
	-	RPGGKKKYR	RPGGKKKYR	RPGGKKKYR	RPGGKKKYR	RPGGKKKYR
	-	NTIATLWCV	-	-	NTIATLWCV	NTIATLWCV
**Novel epitopes**	-	KDCTERQAN	-	-	-	-
	-	TEPTAPPAE	-	-	-	-
	PGGKKKYKM	-	-	-	-	-
	FNCGREGHL	-	-	-	-	-

**Fig 6:**
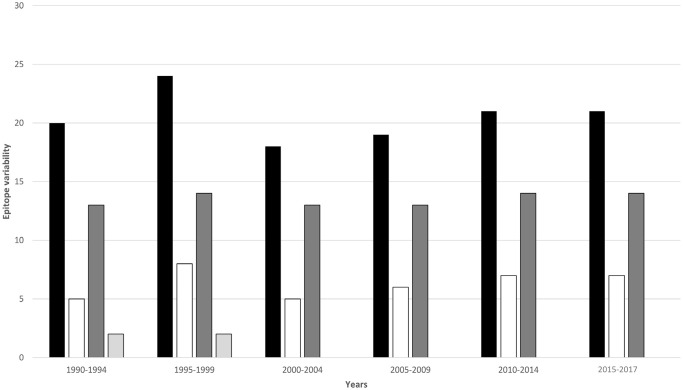
Divergence and evolution of HIV-1 CRF01_AE *gag* CTL epitopes. Bar chart summarizing epitope data for each year group. Black bars show the total number of *gag* epitopes observed for each year group, white bars represent epitope variability (total number of mutations in all epitope sequences in the year group/total number of epitopes in a year group), dark grey bars indicate intermittently recurring epitopes, and light grey bars indicate novel epitopes that were observed in one year group only.

To analyze the time-dependent diversity of *gag* epitopes, the number of CTL epitopes, their HLA restriction pattern, and epitope variability (defined here as the total number of mutations in all epitope sequences in a given year group/ total number of epitopes in a year group) were evaluated. Analysis of HIV-1 CRF01_AE *gag* epitopes showed that the epitope variability increased gradually after 1990, reaching a maximum in the 1995–1999 year group (5.95%), then declined to its minimum in the 2000–2004 year group (3.87%), rose again to reach its maximum (5.95%) in 2010, and sustained till 2017 (Fig 6; Table 1). Meanwhile, the highest number of novel epitopes (n = 4) were present in the 1995–1999 year group, and no novel epitopes were seen after 2000. With regards to the *gag* epitope HLA restriction pattern, unique HLA sequences were predicted almost for each year group. For instance, the CTL epitope RIKCFNCGR present in 1990–1994 year-block was predicted to bind to HLA-A*3302, while a mutation in this epitope changed the epitope sequence to RIKCFNCGK in the 1995–1999, and subsequent year groups, resulting in a change of HLA restriction pattern to HLA-A*0204, HLA-A*1101, and HLA-A3 (S3 Table).

**Fig 7:**
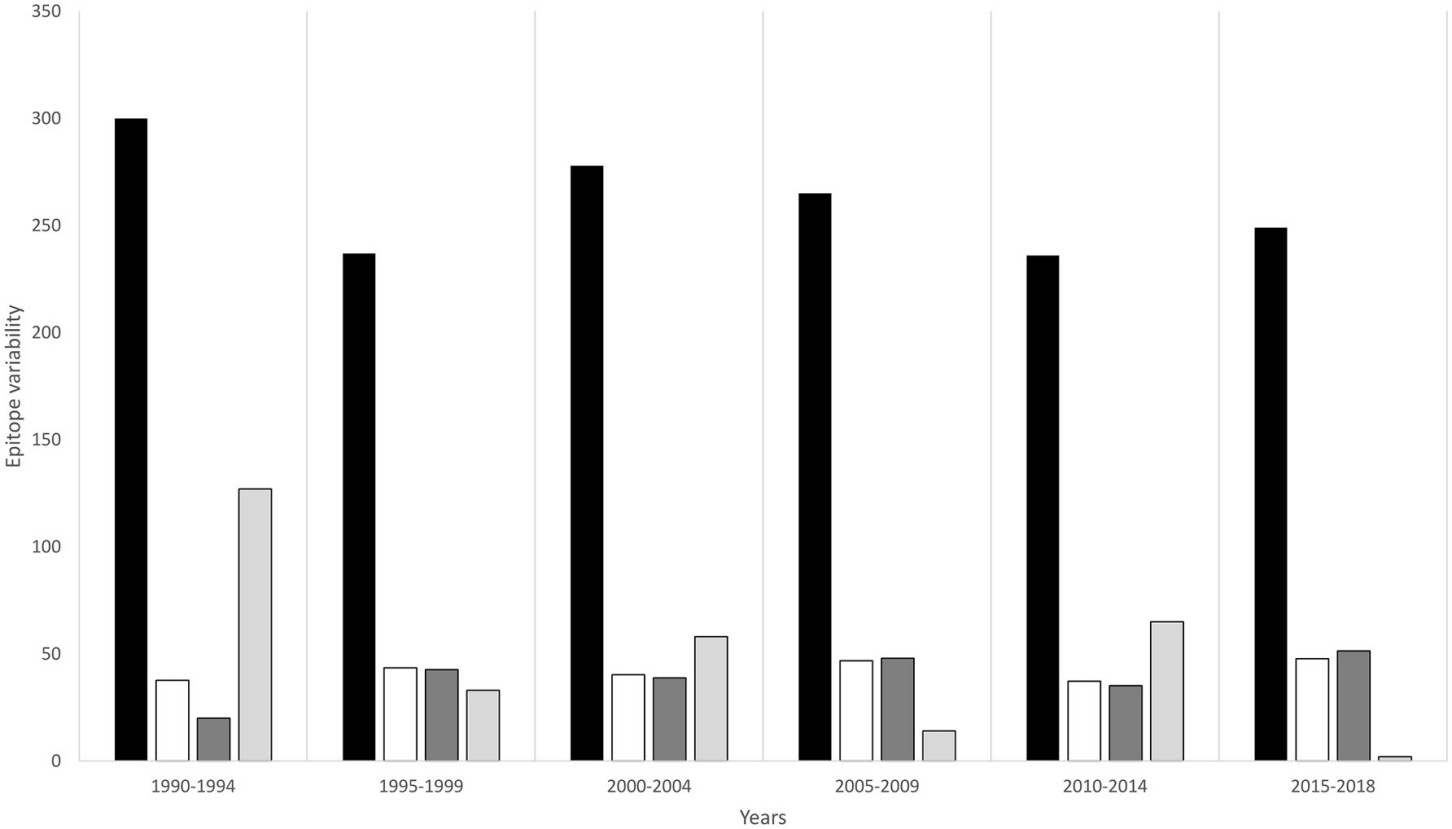
Divergence and evolution of HIV-1 CRF01_AE envelope CTL epitopes. Bar chart summarizing epitope data for each year group. Black bars show the total number of envelope epitopes observed for each year group, white bars represent epitope variability (total number of mutations in all epitope sequences in the year group/total number of epitopes in a year group), dark grey bars indicate intermittently recurring epitopes, and light grey bars indicate novel epitopes that were observed in one year group only.

Since similar to *gag*, HIV-1 *env* is one of the most immunodominant proteins, we also analyzed the time-dependent diversity of *env* epitopes. The *env* epitope variability was found to gradually increase to reach its maximum in the 1995–1999 year group (43.4%), then declined slightly and then rose again to reach its maximum (46.7%) in 2009. After a sudden decline (38.8%) in 2014, variability increased again (51%) in the 2015–2018 year group (Fig 7). The highest number (n = 127) of novel epitopes were present in the 1990–1994 year group, which kept on declining in subsequent years to an all-time low (n = 2) in 2018 (S4 Table).

## Discussion

In this study, using full-length 3105 *gag* sequences between 1990–2017 from the HIV-1 CRF01_AE representing 17 countries submitted to the LANL HIV Sequence Database, we examined the time-dependent evolution of HIV-1 CRF01_AE. Our analysis was specifically focused on the genetic variability and epitope diversity in these gene regions.

The majority of *gag* sequences belonged to Thailand followed by Vietnam and China. Phylogenetic analysis of *gag* sequences showed two prominent clusters of sequences from China and Vietnam. Analysis according to year demonstrated that one cluster contained sequences from 2009 and 2012 while the other contained sequences from 2013, 2010, and 2005. The phylogenetic relationship between the sequences can be explained by the geographical proximity of these countries to one another which allowed for cross-border transmission, once it had permeated the population. This pattern has also been seen previously in studies analyzing the phylogenetic relationship of HIV-1 CRF01_AE in these countries [4, 24, 25]. Similar patterns of spread of epidemics have been seen in the spread of other subtypes such as for HIV-1 Subtype A in Pakistan and Afghanistan [26].

Analysis of the origin of the virus using Bayesian analysis showed that the time to the most recent ancestor of HIV-1 CRF01_AE *gag* sequences was around 1974. Previous studies support these findings and have shown that the first HIV-1 CRF01_AE strain was present in Central Africa in the mid-1970s [3]. After the origin of the virus, the effective population size increased rapidly between 1980–1989 as the virus spread to new populations. This period corresponds to the years after the introduction of the virus in Vietnam (1990) and Thailand (1979–1982) [4, 27]. In our analysis, a decrease in effective population size was seen in effective population size (correlating with an effective number of infections and/or transmission opportunities [23]) of *gag* sequences between 1993–1996. This decline may be a result of the implementation of effective control measures (such as a 100% condom use per sex policy) in countries such as Thailand in the late 1980s [28]. This period of decline was followed by an increase in the effective population size of *gag* sequences between 1996–1998. It was during these years that the virus was introduced to China and began spreading in the country [5]. Previous studies analyzing HIV *gag* have found similar declines in effective population size in the second half of the 1990s followed by an increase in effective population size in the 2000s [5]. The effective population of *gag* sequences remained stable afterward as transmission remained high.

Shannon entropy was used as a measure of time-dependent genetic evolution. It is a quantitative measurement of the uncertainty in a data set i.e. the probability of acquiring mutations in a genomic sequence, where an increase in entropy values reflects a higher probability for acquiring mutations [29]. Shannon entropy has been used as a measure of the variability of HIV-1 in previous studies [30]. A consistent increase in entropy values was seen across year groups. The difference in mean entropy values of the first three year groups with all other year groups was significant (p<0.05), while the difference in mean entropy between the 2000–2005 and 2015–2017 and 2005–2010 and 2010–2015 was statistically insignificant (Fig 4).

The proteasome machinery degrades proteins into peptides of varying lengths, which are then sequentially processed and displayed by the HLA-I molecules to T lymphocytes [31]. Multiple studies have reported that many T cell epitopes are generated via C-terminus cleavage, while certain epitopes are generated via N-terminus cleavage, which is carried out by proteases present in the endoplasmic reticulum [32, 33]. Mutations both within, and in the flanking region (~14 amino acids up- or down-stream of epitope) can affect proteasomal cleavage, thus disrupting T cell generation [34]. In our study, overall, all proteasomal sites were similar among all year groups except for a few places, such as positions 30, 82, 83, and 370, where scores of the sites were different between the year groups. Mutations at positions 30, 82, and 83 resulted in changes in epitope sequences between the year groups, while mutation at the position at 370 resulted in the emergence of new epitopes in 1990–1994, 2000–2004, and 2005–2009 year groups.

CTL epitope variability and HLA binding epitope variability were also used to understand the time-dependent genetic evolution of the virus (S3 Table). Epitope variability for *gag* reached its maximum value between 1995–1999 which corresponds to the rise in effective population size during this period, followed by a decline in the 2000–2004 year group, and a gradual return to its maximum value, which was sustained after 2010 and corresponded to a stabilization of the effective population size. The selection pressure over the years may have resulted in genetic evolution which provided the virus ability to escape host response mechanisms and permeate the population [35, 36]. Similar patterns of variability due to selection pressures have been seen in HIV-1 subtype A [19]. The highest number of novel epitopes were present in the 1990–1994 year group and there were no novel epitopes present after 2000. Stabilization of epitope variability and absence of novel epitopes was consistent with the stabilization of entropy (insignificant difference in mean entropy of last three year groups) and effective population size and may correspond to plans implemented in Thailand to eliminate mother-to-child HIV transmission.

HLA restriction analysis showed binding of certain HLA alleles including HLA-B*51, HLA-B*27, and HLA-B*5801 with unique or mutated epitopes (S3 Table). Previous studies have shown protective effects of restriction with certain HLA alleles against HIV-1 CRF01_AE infections, for example, the presence of HLA-B*57:01, HLA-B*35:05, and HLA-B*51 were seen to reduce disease progression in the Thai population [37, 38]. The presence of restriction with these HLA alleles with unique epitopes may have reduced the spread of HIV in that period. Conversely, HLA alleles such as HLA-B*27, B*57, and B*5801 have been shown to induce protective T cell responses in Caucasian populations, B*13 in African populations, and HLA-B*51 in Asian populations are seen to be ineffective against HIV-1 CRF01_AE in Chinese populations [39, 40]. The lack of protective effects may be responsible for the rapid spread of the virus in China [40].

There were a limited number of sequences available for 1990–1994, and 1995–1999 year groups. A greater number of sequences from these years would have provided further insight into the spread of HIV soon after the origin of a recombinant form. This information will be useful in understanding and responding to recombinant forms of the virus as they originate. A year-wise analysis of epitope sequences from the same populations will further help in deciphering the true nature of this phenomenon and provide a better understanding of the direction in which the HIV-1 epidemics continue to evolve. This information will be crucial in anticipating prevention and control strategies for CRF01_AE -infected patients, especially in populations where the epidemic is newly emerging.

## Supporting information

S1 Table HIV-1 CRF01_AE *gag* number of sequences from each country per year group. (DOCX)

S2 Table HIV-1 CRF01_AE *env* number of sequences from each country per year group. (DOCX)

S3 Table HIV-1 CRF01_AE *gag* mutated and novel epitopes with HLA restriction binding for mutated epitopes. Mutation in the epitope is represented by the color red.
(DOCX)

S4 Table HIV-1 CRF01_AE envelope CTL mutated and novel epitopes. The epitopes are divided in three categories: Novel epitopes (epitopes unique to one year group), intermittently recurring epitopes and mutated epitopes. Mutation(s) in epitope are underlined, while ‘-‘ in the table represents the absence of epitope in a particular year group.
(DOCX)
