# Supplementary material for: Analysis of temporal changes in HIV-1 CRF01_AE gag genetic variability and CD8 T-cell epitope evolution
Source: PLoS One. 2022 May 10;17(5):e0267130. doi: 10.1371/journal.pone.0267130 (PMC9089901; doi:10.1371/journal.pone.0267130)
Supplement: S1 Table — (DOCX) [file pone.0267130.s001.docx]

**Table S1:**

| Year- group | Total sequences | Countries represented in each year-group | Number of sequences |
| --- | --- | --- | --- |
| 1990-94 | 28 | Central African Republic | 3 |
|  |  | Thailand | 19 |
|  |  | Japan | 4 |
|  |  | Indonesia | 2 |
| 1995-99 | 42 | Thailand | 3 |
|  |  | Vietnam | 33 |
|  |  | China | 2 |
|  |  | Japan | 3 |
|  |  | Myanmar | 1 |
| 2000-04 | 445 | Thailand | 423 |
|  |  | China | 20 |
|  |  | Japan | 1 |
|  |  | Hong Kong | 1 |
| 2005-09 | 1517 | Thailand | 1040 |
|  |  | Cyprus | 1 |
|  |  | China | 468 |
|  |  | United Kingdom | 1 |
|  |  | Sweden | 1 |
|  |  | Japan | 1 |
|  |  | United States | 1 |
|  |  | Afghanistan | 1 |
|  |  | Cameroon | 2 |
|  |  | Switzerland | 1 |
| 2010-14 | 986 | Ireland | 1 |
|  |  | United Kingdom | 13 |
|  |  | Thailand | 237 |
|  |  | China | 359 |
|  |  | Sweden | 5 |
|  |  | Switzerland | 3 |
|  |  | Vietnam | 366 |
|  |  | Cameroon | 1 |
|  |  | Japan | 1 |
| 2015-17 | 87 | Philippines | 18 |
|  |  | China | 55 |
|  |  | Thailand | 14 |
