# Supplementary material for: Analysis of temporal changes in HIV-1 CRF01_AE gag genetic variability and CD8 T-cell epitope evolution
Source: PLoS One. 2022 May 10;17(5):e0267130. doi: 10.1371/journal.pone.0267130 (PMC9089901; doi:10.1371/journal.pone.0267130)
Supplement: S2 Table — (DOCX) [file pone.0267130.s002.docx]

**Table S2:** *HIV-1 CRF01_AE* env sequences from each country per year group

| **Year- group** | **Total sequences** | **Countries represented in each year-group** | **Number of sequences** |
| --- | --- | --- | --- |
| 1990-94 | 58 | Central African Republic | 3 |
|  |  | Thailand | 44 |
|  |  | Japan | 3 |
|  |  | Finland | 3 |
|  |  | Cameroon | 2 |
|  |  | Indonesia | 2 |
|  |  | France | 1 |
| 1995-99 | 434 | Thailand | 390 |
|  |  | Vietnam | 33 |
|  |  | China | 3 |
|  |  | France | 1 |
|  |  | Myanmar | 1 |
|  |  | United States of America | 3 |
| 2000-04 | 673 | Thailand | 661 |
|  |  | China | 7 |
|  |  | Japan | 1 |
|  |  | United States of America | 1 |
|  |  | Hong Kong | 1 |
|  |  | Cameroon | 2 |
| 2005-09 | 1970 | Thailand | 1372 |
|  |  | Vietnam | 3 |
|  |  | China | 549 |
|  |  | United Kingdom | 2 |
|  |  | Sweden | 1 |
|  |  | Spain | 1 |
|  |  | United States | 1 |
|  |  | Singapore | 27 |
|  |  | Afghanistan | 1 |
|  |  | Cameroon | 2 |
|  |  | Bulgaria | 11 |
| 2010-14 | 1887 | United Kingdom | 8 |
|  |  | Thailand | 1384 |
|  |  | China | 403 |
|  |  | Sweden | 6 |
|  |  | Vietnam | 21 |
|  |  | Cameroon | 1 |
|  |  | Indonesia | 10 |
|  |  | Iran | 1 |
|  |  | Japan | 1 |
|  |  | Laos | 49 |
|  |  | Myanmar | 1 |
|  |  | Nepal | 1 |
|  |  | Slovenia | 1 |
| 2015-18 | 243 | Philippines | 19 |
|  |  | China | 18 |
|  |  | Thailand | 200 |
|  |  | Belgium | 2 |
|  |  | Vietnam | 4 |
