# Supplementary material for: Analysis of temporal changes in HIV-1 CRF01_AE gag genetic variability and CD8 T-cell epitope evolution
Source: PLoS One. 2022 May 10;17(5):e0267130. doi: 10.1371/journal.pone.0267130 (PMC9089901; doi:10.1371/journal.pone.0267130)
Supplement: S3 Table — Mutation in the epitope is represented by the color red. (DOCX) [file pone.0267130.s003.docx]

**Table S3:**

| **1995-1999** | **HLA restriction** | **2000-2004** | **HLA restriction** | **2005-2009** | **HLA restriction** | **2010-2014** | **HLA restriction** | **2015-2017** | **HLA restriction** |
| --- | --- | --- | --- | --- | --- | --- | --- | --- | --- |
| GGKKKYRMK | HLA-B*2704 | GGKKKYRMK | HLA-B*2704 | GGKKKYRMK | HLA-B*2704 | GGKKKYRMK | HLA-A24 | GGKKKYRMK | HLA-A24 |
|  | HLA-B44 |  | HLA-B44 |  | HLA-B44 |  | HLA-B*2704 |  | HLA-B*2704 |
|  |  |  |  |  |  |  | HLA-B44 |  | HLA-B44 |
| RTEPTAPPA | HLA-A2 | RPEPTAPPA |  | RPEPTAPPA |  | RPEPTAPPA |  | RPEPTAPPA |  |
|  | HLA-B*5801 |  |  |  |  |  |  |  |  |
|  | HLA-B44 |  |  |  |  |  |  |  |  |
| KYRMKHLVW |  | KYRMKHLVW |  | KYRMKHLVW |  | KYRMKHLVW | HLA-A*2402 | KYRMKHLVW | HLA-A*2402 |
| YRMKHLVWA | HLA-A1 | YRMKHLVWA | HLA-A1 | YRMKHLVWA | HLA-A1 | YRMKHLVWA | HLA-A1 | YRMKHLVWA | HLA-A1 |
|  | HLA-B*2702 |  | HLA-B*2702 |  | HLA-B*2702 |  | HLA-B*2702 |  | HLA-B*2702 |
|  | HLA-B14 |  | HLA-B14 |  | HLA-B14 |  | HLA-B14 |  | HLA-B14 |
| ELKSLFNTI |  | ELKSLFNTV | HLA-A*0205 | ELKSLFNTV | HLA-A*0205 | ELKSLFNTI |  | ELKSLFNTI |  |
|  |  |  | HLA-B*5301 |  | HLA-B*5301 |  |  |  |  |
| SLFNTIATL |  | SLFNTVVTL |  | SLFNTVVTL | HLA-A2.1 | SLFNTIATL |  | SLFNTIATL |  |
| DKIEEVQNK | HLA-A*0201 | DKIEEVQNK | HLA-A*0201 | DKIEEVQNK | HLA-A*0201 | DKIEEVQKK | HLA-A*0201 | DKIEEVQKK | HLA-A*0201 |
|  | HLA-A*0205 |  | HLA-A*0205 |  | HLA-A*0205 |  | HLA-B8 |  | HLA-B8 |
|  | HLA-A2.1 |  | HLA-A2.1 |  | HLA-B8 |  |  |  |  |
|  |  |  | HLA-B8 |  |  |  |  |  |  |
| IEEVQNKSQ | | IEEVQNKSQ | | IEEVQNKSQ | | IEEVQKKSQ | HLA-B*2705 | IEEVQKKSQ | HLA-B*2705 |
| MTNNPPIPV | HLA-A*0201 | MTNNPPIPV |  | MTNNPPIPV |  | MTSNPPIPV |  | MTSNPPIPV |  |
|  | HLA-A*0203 |  |  |  |  |  |  |  |  |
|  | HLA-A*0202 |  |  |  |  |  |  |  |  |
|  | HLA-A*0206 |  |  |  |  |  |  |  |  |
|  | HLA-A*1101 |  |  |  |  |  |  |  |  |
|  | HLA-A11 |  |  |  |  |  |  |  |  |
|  | HLA-A*2402 |  |  |  |  |  |  |  |  |
|  | HLA-A3 |  |  |  |  |  |  |  |  |
|  | HLA-A31 |  |  |  |  |  |  |  |  |
|  | HLA-A*0301 |  |  |  |  |  |  |  |  |
|  | HLA-A2.1 |  |  |  |  |  |  |  |  |
|  | HLA-B14 |  |  |  |  |  |  |  |  |
|  | HLA-B27 |  |  |  |  |  |  |  |  |
|  | HLA-B*2705 |  |  |  |  |  |  |  |  |
|  | HLA-A*6801 |  |  |  |  |  |  |  |  |
|  | HLA-A*6802 |  |  |  |  |  |  |  |  |
|  | HLA-B*3501 |  |  |  |  |  |  |  |  |
|  | HLA-B*5101 |  |  |  |  |  |  |  |  |
|  | HLA-B*5301 |  |  |  |  |  |  |  |  |
|  | HLA-B*5401 |  |  |  |  |  |  |  |  |
|  | HLA-B*51 |  |  |  |  |  |  |  |  |
|  | HLA-B7 |  |  |  |  |  |  |  |  |
|  | HLA-B*0702 |  |  |  |  |  |  |  |  |
|  | HLA-B8 |  |  |  |  |  |  |  |  |
| RVLAEAMSH |  | RVLAEAMSQ | HLA-A*1101 | RVLAEAMSQ | HLA-A*1101 | RVLAEAMSQ | HLA-A*1101 | RVLAEAMSQ | HLA-A*0202 |
|  |  |  | HLA-A31 |  | HLA-A31 |  | HLA-A31 |  | HLA-A*0206 |
|  |  |  | HLA-A*0201 |  | HLA-A*0201 |  | HLA-A*0201 |  | HLA-A*1101 |
|  |  |  | HLA-A*0202 |  | HLA-A*0202 |  | HLA-A*0202 |  | HLA-A11 |
|  |  |  | HLA-A*0206 |  | HLA-A*0206 |  | HLA-A*0206 |  | HLA-A3 |
|  |  |  | HLA-A*1101 |  | HLA-A*1101 |  | HLA-A*1101 |  | HLA-A31 |
|  |  |  | HLA-A11 |  | HLA-A11 |  | HLA-A11 |  | HLA-A*0301 |
|  |  |  | HLA-A3 |  | HLA-A3 |  | HLA-A3 |  | HLA-A2.1 |
|  |  |  | HLA-A31 |  | HLA-A31 |  | HLA-A31 |  | HLA-B14 |
|  |  |  | HLA-A*0301 |  | HLA-A*0301 |  | HLA-A*0301 |  | HLA-B27 |
|  |  |  | HLA-A2.1 |  | HLA-A2.1 |  | HLA-A2.1 |  | HLA-B*2705 |
|  |  |  | HLA-B14 |  | HLA-B14 |  | HLA-B14 |  | HLA-A*6801 |
|  |  |  | HLA-B27 |  | HLA-B27 |  | HLA-B27 |  | HLA-A*6802 |
|  |  |  | HLA-B*2705 |  | HLA-B*2705 |  | HLA-B*2705 |  | HLA-B*3501 |
|  |  |  | HLA-A*6801 |  | HLA-A*6801 |  | HLA-A*6801 |  | HLA-B*5101 |
|  |  |  | HLA-A*6802 |  | HLA-A*6802 |  | HLA-A*6802 |  | HLA-B*5301 |
|  |  |  | HLA-B*3501 |  | HLA-B*3501 |  | HLA-B*3501 |  | HLA-B*5401 |
|  |  |  | HLA-B*5101 |  | HLA-B*5101 |  | HLA-B*5101 |  | HLA-B7 |
|  |  |  | HLA-B*5301 |  | HLA-B*5301 |  | HLA-B*5301 |  | HLA-B*0702 |
|  |  |  | HLA-B*5401 |  | HLA-B*5401 |  | HLA-B*5401 |  | HLA-B8 |
|  |  |  | HLA-B7 |  | HLA-B7 |  | HLA-B7 |  | HLA-A*0201 |
|  |  |  | HLA-B*0702 |  | HLA-B*0702 |  | HLA-B*0702 |  | HLA-A*0203 |
|  |  |  | HLA-B8 |  | HLA-B8 |  | HLA-B8 |  | HLA-A*0202 |
| RIKCFNCGK | HLA-A*0204 | RIKCFNCGK | HLA-A*0204 | RIKCFNCGK | HLA-A*0204 | RIKCFNCGK | HLA-A*0205 | RIKCFNCGK | HLA-A*1101 |
|  | HLA-A*1101 |  | HLA-A*1101 |  | HLA-A*1101 |  | HLA-A*0204 |  | HLA-A3 |
|  | HLA-A3 |  | HLA-A3 |  | HLA-A3 |  | HLA-A*1101 |  |  |
| HVQHANIMM | | QAQHANIMM | HLA-B*3501 | QAQHANIMM | HLA-B*3501 | QVQQTNIMM |  | QVQQTNIMM |  |
|  |  |  | HLA-B*5103 |  | HLA-B*5103 |  |  |  |  |
| KKKYRMKHL |  | KKKYRMKHL |  | KKKYRMKHL |  | KKKYRMKHL |  | KKKYRMKHL |  |
|  |  |  |  |  |  |  |  |  |  |
|  |  |  |  |  |  |  |  |  |  |
|  |  |  |  |  |  |  |  |  |  |
| VLAEAMSHV | HLA-A*0201 |  |  |  |  | VLAEAMSQV | HLA-A*0201 | VLAEAMSQV | HLA-A*0206 |
|  | HLA-A*0203 |  |  |  |  |  | HLA-A*0203 |  | HLA-A*1101 |
|  | HLA-A*0202 |  |  |  |  |  | HLA-A*0202 |  | HLA-A11 |
|  | HLA-A*0206 |  |  |  |  |  | HLA-A*0206 |  | HLA-A*2402 |
|  | HLA-A*1101 |  |  |  |  |  | HLA-A*1101 |  | HLA-A3 |
|  | HLA-A11 |  |  |  |  |  | HLA-A11 |  | HLA-A31 |
|  | HLA-A*2402 |  |  |  |  |  | HLA-A*2402 |  | HLA-A*0301 |
|  | HLA-A3 |  |  |  |  |  | HLA-A3 |  | HLA-A2.1 |
|  | HLA-A31 |  |  |  |  |  | HLA-A31 |  | HLA-B14 |
|  | HLA-A*0301 |  |  |  |  |  | HLA-A*0301 |  | HLA-B27 |
|  | HLA-A2.1 |  |  |  |  |  | HLA-A2.1 |  | HLA-B*2705 |
|  | HLA-B14 |  |  |  |  |  | HLA-B14 |  | HLA-A*6801 |
|  | HLA-B27 |  |  |  |  |  | HLA-B27 |  | HLA-A*6802 |
|  | HLA-B*2705 |  |  |  |  |  | HLA-B*2705 |  | HLA-B*3501 |
|  | HLA-A*6801 |  |  |  |  |  | HLA-A*6801 |  | HLA-B*5101 |
|  | HLA-A*6802 |  |  |  |  |  | HLA-A*6802 |  | HLA-B*5301 |
|  | HLA-B*3501 |  |  |  |  |  | HLA-B*3501 |  | HLA-B*5401 |
|  | HLA-B*5101 |  |  |  |  |  | HLA-B*5101 |  | HLA-B*51 |
|  | HLA-B*5301 |  |  |  |  |  | HLA-B*5301 |  | HLA-B7 |
|  | HLA-B*5401 |  |  |  |  |  | HLA-B*5401 |  | HLA-B*0702 |
|  | HLA-B*51 |  |  |  |  |  | HLA-B*51 |  | HLA-B8 |
|  | HLA-B7 |  |  |  |  |  | HLA-B7 |  | HLA-A2 |
|  | HLA-B*0702 |  |  |  |  |  | HLA-B*0702 |  | HLA-A*0205 |
|  | HLA-B8 |  |  |  |  |  | HLA-B8 |  | HLA-A*0205 |
|  | HLA-A2 |  |  |  |  |  | HLA-A2 |  | HLA-A*3101 |
|  | HLA-A*0205 |  |  |  |  |  | HLA-A*0205 |  | HLA-A*0204 |
| KDCTERQAN | HLA-B*3701 |  |  |  |  |  |  |  |  |
| TEPTAPPAE | HLA-A24 |  |  |  |  |  |  |  |  |
|  | HLA-A*2402 |  |  |  |  |  |  |  |  |
| NWGMGEEIT | HLA-A*0201 |  |  |  |  | NWGMGEEIT |  | NWGMGEEIT |  |
|  | HLA-B*5102 |  |  |  |  |  |  |  |  |
|  | HLA-B*5103 |  |  |  |  |  |  |  |  |
|  |  | AQHANIMMQ | HLA-B*2705 | AQHANIMMQ | HLA-B*2705 |  |  |  |  |
|  |  | SQAQHANIM | HLA-B*3902 | SQAQHANIM | HLA-B*3902 |  |  |  |  |
|  |  |  | HLA-B*5201 |  | HLA-B*5201 |  |  |  |  |
|  |  |  |  |  |  | GGPSHKARV | HLA-B*5201 | GGPSHKARV | HLA-B*5201 |
|  |  |  |  |  |  |  | HLA-B*5101 |  | HLA-B*5101 |
|  |  |  |  |  |  |  | HLA-B*5102 |  | HLA-B*5102 |
|  |  |  |  |  |  |  | HLA-B*5103 |  | HLA-B*5103 |
| KIEEVQNKS | HLA-A1 | KIEEVQNKS | HLA-A*0203 | KIEEVQNKS | HLA-A*0203 |  |  |  |  |
|  |  |  | HLA-A1 |  | HLA-A1 |  |  |  |  |
| DIAGTTSTL | HLA-B*2902 | DIAGTTSTL | HLA-B*3801 | DIAGTTSTL | HLA-B*3801 |  |  |  |  |
| EELKSLFNT | HLA-B40 |  |  |  |  | EELKSLFNT | HLA-B40 | EELKSLFNT | HLA-B40 |
|  | HLA-B*4403 |  |  |  |  |  | HLA-B*4403 |  | HLA-B*4403 |
|  | HLA-B60 |  |  |  |  |  | HLA-B60 |  | HLA-B60 |
|  | HLA-B61 |  |  |  |  |  | HLA-B61 |  | HLA-B61 |
|  | HLA-B44 |  |  |  |  |  | HLA-B44 |  | HLA-B44 |
| KSLFNTIAT | HLA-A2 |  |  |  |  | KSLFNTIAT | HLA-B*5102 | KSLFNTIAT | HLA-B*5102 |
|  | HLA-A*0206 |  |  |  |  |  | HLA-A2 |  | HLA-A2 |
|  |  |  |  |  |  |  | HLA-A*0206 |  | HLA-A*0206 |
| ATLWCVHQR | HLA-A*3101 |  |  | ATLWCVHQR | HLA-A*3101 | ATLWCVHQR | HLA-A*3101 | ATLWCVHQR | HLA-A*3101 |
|  | HLA-A*3302 |  |  |  | HLA-A*3302 |  | HLA-A*3302 |  | HLA-A*3302 |
|  | HLA-A68.1 |  |  |  | HLA-A68.1 |  | HLA-A68.1 |  | HLA-A68.1 |
| RPGGKKKYR | HLA-A68.1 | RPGGKKKYR | HLA-A68.1 | RPGGKKKYR | HLA-A68.1 | RPGGKKKYR | HLA-A68.1 | RPGGKKKYR | HLA-A68.1 |
|  | HLA-B*0702 |  | HLA-B*0702 |  | HLA-B*0702 |  | HLA-B*0702 |  | HLA-B*0702 |
| NTIATLWCV | HLA-A*0201 |  |  |  |  | NTIATLWCV | HLA-A*0201 | NTIATLWCV | HLA-A*0201 |
|  | HLA-A*0203 |  |  |  |  |  | HLA-A*0203 |  | HLA-A*0203 |
|  | HLA-A*0202 |  |  |  |  |  | HLA-A*0202 |  | HLA-A*0202 |
|  | HLA-A*0206 |  |  |  |  |  | HLA-A*0206 |  | HLA-A*0206 |
|  | HLA-A*1101 |  |  |  |  |  | HLA-A*1101 |  | HLA-A*1101 |
|  | HLA-A11 |  |  |  |  |  | HLA-A11 |  | HLA-A11 |
|  | HLA-A*2402 |  |  |  |  |  | HLA-A*2402 |  | HLA-A*2402 |
|  | HLA-A3 |  |  |  |  |  | HLA-A3 |  | HLA-A3 |
|  | HLA-A31 |  |  |  |  |  | HLA-A31 |  | HLA-A31 |
|  | HLA-A*0301 |  |  |  |  |  | HLA-A*0301 |  | HLA-A*0301 |
|  | HLA-A2.1 |  |  |  |  |  | HLA-A2.1 |  | HLA-A2.1 |
|  | HLA-B14 |  |  |  |  |  | HLA-B14 |  | HLA-B14 |
|  | HLA-B27 |  |  |  |  |  | HLA-B27 |  | HLA-B27 |
|  | HLA-B*2705 |  |  |  |  |  | HLA-B*2705 |  | HLA-B*2705 |
|  | HLA-A*6801 |  |  |  |  |  | HLA-A*6801 |  | HLA-A*6801 |
|  | HLA-A*6802 |  |  |  |  |  | HLA-A*6802 |  | HLA-A*6802 |
|  | HLA-B*3501 |  |  |  |  |  | HLA-B*3501 |  | HLA-B*3501 |
|  | HLA-B*5101 |  |  |  |  |  | HLA-B*5101 |  | HLA-B*5101 |
|  | HLA-B*5301 |  |  |  |  |  | HLA-B*5301 |  | HLA-B*5301 |
|  | HLA-B*5401 |  |  |  |  |  | HLA-B*5401 |  | HLA-B*5401 |
|  | HLA-B*51 |  |  |  |  |  | HLA-B*51 |  | HLA-B*51 |
|  | HLA-B7 |  |  |  |  |  | HLA-B7 |  | HLA-B7 |
|  | HLA-B*0702 |  |  |  |  |  | HLA-B*0702 |  | HLA-B*0702 |
|  | HLA-B8 |  |  |  |  |  | HLA-B8 |  | HLA-B8 |
|  | HLA-A2 |  |  |  |  |  | HLA-A2 |  | HLA-A2 |
|  | HLA-A*0201 |  |  |  |  |  | HLA-A*0201 |  | HLA-A*0201 |
