# Supplementary material for: Analysis of temporal changes in HIV-1 CRF01_AE gag genetic variability and CD8 T-cell epitope evolution
Source: PLoS One. 2022 May 10;17(5):e0267130. doi: 10.1371/journal.pone.0267130 (PMC9089901; doi:10.1371/journal.pone.0267130)
Supplement: S4 Table — The epitopes are divided in three categories: Novel epitopes (epitopes unique to one year group), intermittently recurring epitopes and mutated epitopes. Mutation(s) in epitope are underlined, while ‘-‘ in the table represents the absence of epitope in a particular year group. (DOCX) [file pone.0267130.s004.docx]

**Table S4:**

|  | **1990-1994** | **1995-1999** | **2000-2004** | **2005-2009** | **2010-2014** | **2015-2018** |
| --- | --- | --- | --- | --- | --- | --- |
| **Mutated epitopes** | AKRRVVRKR | AKRRVVRKR | AKRRVVRKR | AKRRVVERE | AKRRVVERE | AKRRVVERE |
|  | AVGIGAMIF | AVGIGAMIF | AVGIGAMIF | AVGIGAMIF | AVGLGAMIF | AVGIGAMIF |
|  | AYCINGTKW | AYCINGTKW | AYCINGTKW | AYCEVNGTK | AYCEINGTE | AYCEVNGTK |
|  | DPNPQEIHL | DPNPQEIHL | DPNPQEIHL | DPNPQEIHL | DPNPQEIYL | DPNPQEIHL |
|  | GDIRKAYCI | GDIRKAYCI | GDIRKAYCI | GDIRKAYCE | GDIRKAYCE | GDIRKAYCE |
|  | GIKQLQARV | GIKQLQARV | GIKQLQARV | GIKQLQARI | GIKQLQARV | GIKQLQARI |
|  | GWTDRVIVA | GWTDRVIVA | GWTDRVIEV | GWTDRVIEV | GWTDRVIEI | GWTDRVIEV |
|  | HQRPDRPRI | HQRPDRPRI | HQRPDRPRI | HQREHDRPE | HQRGPDRPE | HQREHDRPE |
|  | HRLRDFILI | HRLRDFILI | HRLRDFILI | HRLRDLILI | HRLRDLISI | HRLRDLILI |
|  | ILKCNDKNF | ILKCNDKNF | ILKCNDKNF | ILKCNDKNF | ILKCNNKTF | ILKCNDKNF |
|  | IRQGLRALL | IRQGLRALL | IRQGLRALL | IRQGLERAL | IRQGLERAL | IRQGLERAL |
|  | ISNYTNQIY | ISNYTNQIY | ISNYTNQIY | ISNYTNQIY | ISNYTGQIY | ISNYTNQIY |
|  | ITNWLWYIK | ITNWLWYIK | ITNWLWYIK | ITRWLWYIK | ITRWLWYIK | ITRWLWYIK |
|  | KAHTEVHNV | KAHTEVHNV | KAHTEVHNV | KAHETEVHN | KAHETEVHN | KAHETEVHN |
|  | KRAVGIGAM | KRAVGIGAM | KRAVGIGAM | KRAVGIGAM | KRAVGLGAM | KRAVGIGAM |
|  | KRRVVRKRA | KRRVVRKRA | KRRVVRKRA | KRRVVEREK | KRRVVEREK | KRRVVEREK |
|  | NLTNNAKTI | NLTNNAKTI | NLTNNAKTI | NLTNNAKTI | NLTDNTKTI | NLTNNAKTI |
|  | NVTENFNMW | NVTENFNMW | NVTENFNMW | NVTENFNMW | NVTEDFNMW | NVTENFNMW |
|  | QLQARVLAV | QLQARVLAV | QLQARVLAV | QLQARILAV | QLQARVLAV | QLQARVLAV |
|  | QNQQDRNEK | QNQQDRNKD | QNQQDRNKD | QNQQDRNEK | QNQQDRNEK | QNQQDRNEL |
|  | RAKRRVVRK | RAKRRVVRK | RAKRRVVRK | RAKRRVVER | RAKRRVVER | RAKRRVVER |
|  | RAVGIGAMI | RAVGIGAMI | RAVGIGAMI | RAVGIGAMI | RAVGLGAMI | RAVGIGAMI |
|  | RLRDLILIA | RLRDLILIA | RLRDLILIA | RLRDLILIA | RLRDLISIV | RLRDLILIA |
|  | RSVRLVSGF | RSVRLVSGF | RSVRLVSGF | RSVRLVNGF | RSVRLVSGF | RSVRLVNGF |
|  | RLVSGFLAL | RLVSGFLAL | RLVSGFLAL | RLVNGFLAL | RLVSGFLAL | RLVNGFLAL |
|  | SITLTVQAR | SITLTVQAR | SITLTVQAR | SITLTVQAR | SITLTVHAR | SITLTVQAR |
|  | STMGAASIT | STWSNKSIT | STMGAASIT | STMGAASIT | STMGAASIT | STMGAASIT |
|  | SYHRLRDFI | SYHRLRDFI | SYHRLRDFI | SYHRLRDLI | SYHRLRDLI | SYHRLRDLI |
|  | TLNCTNANL | TLNCTNANL | TLNCTNANL | TLNCTNANL | TLNCNDATL | TLNCTNANL |
|  | TLTVQARQL | TLTVQARQL | TLTVQARQL | TLTVQARQL | TLTVHARQL | TLTVQARQL |
|  | YAPPISGRI | YAPPISGRI | YAPPISGSI | YAPPISGRI | YAPPISGSI | YAPAGFAI |
|  | LVSGFLALA | LVSGFLALA | LVSGFLALA | LVNGFLALV | LVSGFLALA | LVNGFLALV |
|  | DRSVRLVSG | - | DRSVRLVSG | DRSVRLVNG | DRSVRLVSG | DRSVRLVNG |
|  | ERYLKDQKF | ERYLKDQKF | - | ERYLKDQKF | ERYLKDQKL | ERYLKDQKF |
|  | FFYCNTTQL | - | FFYCNTTQL | FFYCNTSQL | FFYCNTTKL | FFYCNTSQL |
|  | RVLAVERYL | RVLAVERYL | RVLAVRYLK | - | RVLAVERYL | RVLAVEQAA |
|  | FNCRGFFYC | FNCRGFFYC | FNCRGFFYC | FNCRGEFFY | - | FNCRGEFFY |
|  | FQPPSGGDL | FQPPSGGDL | - | FQIPTHHQR | FQTPTHHQR | FQIPTHHQR |
|  | FYCNTTQLF | FYCNTTQLF | FYCNTTQLF | FYCNTSQLF | - | FYCNTSQLF |
|  | GLRRGWEGL | GLRRGWGLK | GLRRGWGLK | GLRRGWESL | GLRRGCSGKI | - |
|  | IHLNVTENF | IHLNVTENF | IHLNVTENF | IHLENVTEN | - | IHLENVTEN |
|  | - | ILPCKIKQI | ILPCRIKQI | ILPCRIKQI | ILPCRIKQI | ILPCRIKQI |
|  | KAYCINGTK | KAYCINGTK | KAYCINGTK | KAYCEVNGT | - | KAYCEVNGT |
|  | KDQKFLGLW | - | KDQKFLGLW | KDQKFLGLW | KDQKLLGLW | KDQKFLGLW |
|  | KSVINCTRP | KSVINCTRP | KSVINCTRP | KSVEINCTR | - | KSVEINCTR |
|  | KYLGNLLLY | KYLGNLLLY | KYLGNLLLY | KYLGNLLFY | - | KYLGNLLFY |
|  | LRDFILIAA | LRDFILIAA | LRDLILIAA | LRDLILIAA | - | LRDLILIAA |
|  | LTVQARQLL | - | LTVQARQLL | LTVQARQLL | LTVHARQLL | LTVQARQLL |
|  | MVEQMQEDV | MVEQMQEDV | - | MVEQMQEDV | MVEQHEDVI | MVEQMQEDV |
|  | NLLRAIEAQ | - | NLLRAIAQQ | NLLRAIEAQ | NLLRAIEAQ | NLLRAIEAQ |
|  | MYAPPISGR | MYAPPISGR | MYAPPISGI | MYAPPISGR | - | MYAPPISGR |
|  | NWFDITNWL | - | NWLWYIKIF | NWFDITRWL | NWFDITRWL | NWFDITRWL |
|  | PPISGRINC | PPISGRINC | PPISGIINC | PPISGRINC | - | PPISGRINC |
|  | - | SQNQQDRNK | SQNQQDRNK | SQNQQDRNE | SQNQQDKNE | SQNQQDRNE |
|  | TRAKRRVVR | TRAKRRVVR | TRAKRRVVR | TRTRMTIGP | - | TRTRMTIGP |
|  | WNNMTWIWR | - | WNNMTWIWR | WNWFDITRW | WNWFDITRW | WNWFDITRW |
|  | RRGWEGLKY | RRGWGLKYL | RRGWGLKYL | RRGWESLKY | - | RRGWESLKY |
|  | RTGDIIGDI | RTGDIIGDI | RTGDIIGDI | RTGEIIGDI | - | RTGEIIGDI |
|  | ARVLAVERY | ARVLAVERY | ARVLAVRYL | - | ARVLAVERY | - |
|  | QQDRNEKDL | QQDRNEKDL | QQDRNKDLL | - | QQDRNKDLL | - |
|  | - | - | RIKQIINMW | RIKQIINMW | RIKQIIRMW | RIKQIIRMW |
|  | GLKYLGNLL | GLKYLGNLL | GLKYLGNLL | - | GLKYLGSIL | - |
|  | DRVIEVAQGA | DRVIEVAQRA | DRVIEVAQR | - | DRVIEIVQI | - |
|  | HFNCRGFFY | HFNCRGFFY | HFNCRGFFY | HFNCRGEFF | - | - |
|  | IGPGQVFYR | IGPGQVFYR | IGPGQVFYR | - | IGPGQAFYR | - |
|  | MHHFNCRGF | MHHFNCRGF | - | MHHFNCRGE | - | MHHFNCRGE |
|  | ANLTNVNNT | - | ANLTNNNTT | ANLTNVTNT | - | ANLTNVTNT |
|  | - | - | AASITLTVQ | AASITLTVQ | AASITLTVH | AASITLTVQ |
|  | CRGFFYCNT | - | CRGFFYCNT | CRGEFFYCN | - | CRGEFFYCN |
|  | - | - | DITNWLWYI | DITRWLWYI | DITRWLWYI | DITRWLWYI |
|  | EISNYTNQI | - | - | EISNYTNQI | EISNYTGQI | EISNYTNQI |
|  | - | ELKISAISL | - | ELKISAISL | ELKTSAISL | ELKISAISL |
|  | SENLTNNAK | SENLTNNAK | - | SENLTNNAK | SENLTDNTK | - |
|  | - | NPQEIHLNV | NPQEIHLNV | NPQEIHLEN | - | NPQEIHLEN |
|  | RAIEAQQHL | - | RAIEAQQHM | RAIEAQQHM | RAIEAQQHM | - |
|  | - | SFQTPTHHQ | SFQIPTHHQ | SFQTPTHHQ | SFQIPTHHQ | - |
|  | - | - | THHQRPDRP | THHQREHDR | THHQRGPDR | THHQRGPDR |
|  | - | - | TWSNKSYIW | TWTEWEREI | TWVQWEREI | TWTEWEREI |
|  | - | - | YLGNLLLYW | YLGNLLFYW | YLGSILSYW | YLGNLLFYW |
|  | - | LYKYKVVQI | LYKYKVVQI | LYKYKVVEI | - | LYKYKVVEI |
|  | YTNQIYILT | - | YTNQIYILT | YTNQIYNIL | - | YTNQIYNIL |
| **Intermittently recurring epitopes** | ASDNLWVTV | ASDNLWVTV | ASDNLWVTV | ASDNLWVTV | - | ASDNLWVTV |
|  | CVTLNCTNA | CVTLNCTNA | CVTLNCTNA | CVTLNCTNA | - | CVTLNCTNA |
|  | DKKQKVHAL | DKKQKVHAL | DKKQKVHAL | DKKQKVHAL | - | DKKQKVHAL |
|  | DNLWVTVYY | DNLWVTVYY | DNLWVTVYY | DNLWVTVYY | - | DNLWVTVYY |
|  | FLGAAGSTM | - | FLGAAGSTM | FLGAAGSTM | FLGAAGSTM | FLGAAGSTM |
|  | IKIFIMIVG | IKIFIMIVG | IKIFIMIVG | IKIFIMIVG | - | IKIFIMIVG |
|  | KLTPLCVTL | KLTPLCVTL | KLTPLCVTL | KLTPLCVTL | - | KLTPLCVTL |
|  | KVHALFYKL | KVHALFYKL | KVHALFYKL | KVHALFYKL | - | KVHALFYKL |
|  | LFSYHRLRD | - | LFSYHRLRD | LFSYHRLRD | LFSYHRLRD | LFSYHRLRD |
|  | - | LGAAGSTMG | LGAAGSTMG | LGAAGSTMG | LGAAGSTMG | LGAAGSTMG |
|  | - | LQLTVWGIK | LQLTVWGIK | LQLTVWGIK | LQLTVWGIK | LQLTVWGIK |
|  | LPCKIKQII | - | LPCRIKQII | LPCRIKQII | LPCRIKQII | LPCRIKQII |
|  | LRIIFAVLS | - | LRIIFAVLS | LRIIFAVLS | LRIIFAVLS | LRIIFAVLS |
|  | LWGCSGKII | LWGCSGKII | - | LWGCSGKII | LWGCSGKII | LWGCSGKII |
|  | LRSLCLFSY | - | LRSLCLFSY | LRSLCLFSY | LRSLCLFSY | LRSLCLFSY |
|  | NAKTIIVHL | NAKTIIVHL | NAKTIIVHL | NAKTIIVHL | - | NAKTIIVHL |
|  | NFNMWKNNM | NFNMWKNNM | NFNMWKNNM | NFNMWKNNM | - | NFNMWKNNM |
|  | NVSSVQCTH | NVSSVQCTH | NVSSVQCTH | NVSSVQCTH | - | NVSSVQCTH |
|  | VYYGVPVWR | VYYGVPVWR | VYYGVPVWR | VYYGVPVWR | - | VYYGVPVWR |
|  | VERYLKDQK | VERYLKDQK | - | VERYLKDQK | VERYLKDQK | VERYLKDQK |
|  | VISLWDQSL | VISLWDQSL | VISLWDQSL | VISLWDQSL | - | VISLWDQSL |
|  | SASDNLWVT | SASDNLWVT | SASDNLWVT | SASDNLWVT | - | SASDNLWVT |
|  | - | RPGGGNIKD | RPGGGNIKD | RPGGGNIKD | RPGGGNIKD | RPGGGNIKD |
|  | RLINCNTSV | RLINCNTSV | RLINCNTSV | RLINCNTSV | - | RLINCNTSV |
|  | QAMYAPPIS | - | QAMYAPPIS | QAMYAPPIS | QAMYAPPIS | QAMYAPPIS |
|  | AVAGWTDRV | AVAGWTDRV | AVAGWTDRV | - | AVAGWTDRV | - |
|  | KNFNGTGPC | KNFNGTGPC | - | KNFNGTGPC | - | KNFNGTGPC |
|  | QAYALFYKL | QAYALFYKL | QAYALFYKL | - | QAYALFYKL | - |
|  | QSLKPCVKL | - | QSLKPCVKL | QSLKPCVKL | - | QSLKPCVKL |
|  | GRDRSVRLV | GRDRSVRLV | GRDRSVRLV | - | GRDRSVRLV | - |
|  | GRINCVSNI | GRINCVSNI | - | GRINCVSNI | - | GRINCVSNI |
|  | - | KQIINMWQG | KQIINMWQG | KQIINMWQG | - | KQIINMWQG |
|  | KQKVHALFY | KQKVHALFY | - | KQKVHALFY | - | KQKVHALFY |
|  | FLGLWGCSG | FLGLWGCSG | - | FLGLWGCSG | - | FLGLWGCSG |
|  | GFLGAAGST | - | - | GFLGAAGST | GFLGAAGST | GFLGAAGST |
|  | - | IIFQPPSGG | IIFQPPSGG | IIFQPPSGG | - | IIFQPPSGG |
|  | IIVHLNKSV | IIVHLNKSV | - | IIVHLNKSV | - | IIVHLNKSV |
|  | LGLWGCSGK | LGLWGCSGK | - | LGLWGCSGK | LGLWGCSGK | - |
|  | PVVSTQLLL | PVVSTQLLL | PVVSTQLLL | - | PVVSTQLLL | - |
|  | PISGRINCV | PISGRINCV | - | PISGRINCV | - | PISGRINCV |
|  | KTQMNWPNL | KTQMNWPNL | KTQMNWPNL | - | - | KTQMNWPNL |
|  | GNITDEVRN | GNITDEVRN | - | GNITDEVRN | - | GNITDEVRN |
|  | CSGKIICTT | - | CSGKIICTT | CSGKIICTT | - | CSGKIICTT |
|  | GGLIGLRII | - | - | GGLIGLRII | GGLIGLRII | GGLIGLRII |
|  | TAVPWNSTW | TAVPWNSTW | TAVPWNSTW | - | TAVPWNSTW | - |
|  | RDADTTLFC | RDADTTLFC | RDADTTLFC | RDADTTLFC | - | - |
|  | - | RQLLSGIVQ | - | RQLLSGIVQ | RQLLSGIVQ | RQLLSGIVQ |
|  | - | - | WTDRVIEVA | WTDRVIEVA | WTDRVIEIVA | WTDRVIEVA |
|  | - | - | TIILPCRIK | TIILPCRIK | TIILPCRIK | TIILPCRIK |
|  | - | WNSTWSNKS | - | WNSTWSNKS | WNSTWSNKS | WNSTWSNKS |
|  | - | WKWGTLILG | WKWGTLILG | WKWGTLILG | - | WKWGTLILG |
|  | YKVVEIEPL | - | - | YKVVEIEPL | YKVVEIEPL | YKVVEIEPL |
|  | GVPVWRDAD | - | - | GVPVWRDAD | GVPVWRDAD | GVPVWRDAD |
|  | GYSPLSFQI | - | - | GYSPLSFQI | GYSPLSFQI | GYSPLSFQI |
|  | - | RNKDLLLDK | RNKDLLLDK | RNKDLLLDK | - | RNKDLLLDK |
|  | AISLLDATA | AISLLDATA | AISLLDATA | - | - | - |
|  | AKAHTEVHN | AKAHTEVHN | AKAHTEVHN | - | - | - |
|  | AVPWNSTWS | - | AVPWNSTWS | - | AVPWNSTWS | - |
|  | ALAWDDLRS | - | ALAWDDLRS | - | ALAWDDLRS | - |
|  | - | - | - | CASDAKAHE | CASDAKAHE | CASDAKAHE |
|  | CINGTKWNV | CINGTKWNV | CINGTKWNV | - | - | - |
|  | CKIKQIINM | CKIKQIINM | CKIKQIINM | - | - | - |
|  | - | - | - | CNDKNFNGT | CNDKNFNGT | CNDKNFNGT |
|  | DKWASLWNW | DKWASLWNW | DKWASLWNW | - | - | - |
|  | DPIPIHYCT | DPIPIHYCT | DPIPIHYCT | - | - | - |
|  | CTNANLTNV | - | - | CTNANLTNV | - | CTNANLTNV |
|  | - | - | - | DLLELDKWA | DLLELDKWA | DLLELDKWA |
|  | - | - | - | EAQQHMLQL | EAQQHMLQL | EAQQHMLQL |
|  | - | - | - | CRIKQIINM | CRIKQIINM | CRIKQIINM |
|  | - | - | - | ARTVELLGR | ARTVELLGR | ARTVELLGR |
|  | - | - | - | EEDGGEQDR | EEDGGEQDR | EEDGGEQDR |
|  | - | - | - | EIIIRSENL | EIIIRSENL | EIIIRSENL |
|  | - | - | - | EIWNNMTWT | EIWNNMTWT | EIWNNMTWT |
|  | - | - | - | ELDKWASLW | ELDKWASLW | ELDKWASLW |
|  | - | - | - | ENVTENFNM | ENVTENFNM | ENVTENFNM |
|  | - | - | - | FCASDAKAH | FCASDAKAH | FCASDAKAH |
|  | - | - | - | FDITRWLWY | FDITRWLWY | FDITRWLWY |
|  | - | - | - | GNIKDNWRS | GNIKDNWRS | GNIKDNWRS |
|  | - | - | - | GRSSCKGLR | GRSSCKGLR | GRSSCKGLR |
|  | GSLAIIIRS | GSLAIIIRS | GSLAIIIRS | - | - | - |
|  | - | - | - | HETEVHNVW | HETEVHNVW | HETEVHNVW |
|  | - | - | - | GSLAEEEII | GSLAEEEII | GSLAEEEII |
|  | - | - | - | IFAVLSIVN | IFAVLSIVN | IFAVLSIVN |
|  | - | IGNITDEVR | - | IGNITDEVR | - | IGNITDEVR |
|  | IKQIINMWQ | - | - | IKQIINMWQ | - | IKQIINMWQ |
|  | INCTRPSTN | INCTRPSTN | - | - | INCTRPSTN | - |
|  | INCVSNITG | INCVSNITG | INCVSNITG | - | - | - |
|  | - | IILPCRIKQ | IILPCRIKQ | - | IILPCRIKQ |  |
|  | - | - | KFLGLWGCS | KFLGLWGCS | - | KFLGLWGCS |
|  | GEIIGDIRK | - | - | GEIIGDIRK | - | GEIIGDIRK |
|  | PIHYCTPAG | - | PIHYCTPAG | - | PIHYCTPAG | - |
|  | - | NGSNIIGNI | NGSNIIGNI | - | NGSNIIGNI | - |
|  | - | NITDEVRNC | - | NITDEVRNC | - | NITDEVRNC |
|  | - | NKSVEINCT | NKSVEINCT | - | - | NKSVEINCT |
|  | - | NMWQGVGQA | - | NMWQGVGQA | - | NMWQGVGQA |
|  | - | - | - | LENVTENFN | LENVTENFN | LENVTENFN |
|  | - | - | LGNLLLYWG | LGNLLLYWG | - | LGNLLLYWG |
|  | - | - | - | LGRSSCKGL | LGRSSCKGL | LGRSSCKGL |
|  | IAARTVELL | - | - | IAARTVELL | - | IAARTVELL |
|  | PLGIAPTRA | - | - | PLGIAPTRA | PLGIAPTRA | - |
|  | FNGTGPCKN | FNGTGPCKN | - | - | - | FNGTGPCKN |
|  | GEQGRDRSV | GEQGRDRSV | - | GEQGRDRSV | - | - |
|  | LVIICSASD | LVIICSASD | LVIICSASD | - | - | - |
|  | SELYKYKVV | - | - | SELYKYKVV | SELYKYKVV | - |
|  | RPSNNTRTS | RPSNNTRTS | - | RPSNNTRTS | - | - |
|  | - | - | RVIEVAQRA | RVIEVAQRA | RVIEVAQRA | - |
|  | KWASLWNWF | KWASLWNWF | KWASLWNWF | - | - | - |
|  | KDNWRSELY | KDNWRSELY | KDNWRSELY | - | - |  |
|  | - | - | - | PKISFDPIP | PKISFDPIP | PKISFDPIP |
|  | - | - | - | PVWRDADTT | PVWRDADTT | PVWRDADTT |
|  | PNLWKWGTL | PNLWKWGTL | PNLWKWGTL | - | - | - |
|  | PNPQEIHLN | PNPQEIHLN | PNPQEIHLN | - | - | - |
|  | - | - | - | PERTEEDGG | PERTEEDGG | PERTEEDGG |
|  | - |  | LKQVAKKLK | LKQVAKKLK | - | LKQVAKKLK |
|  | - | LLDATAIAV | LLDATAIAV | - | LLDATAIAV | - |
|  | - | LLGHSSLKG | LLGHSSLKG | - | LLGHSSLKG | - |
|  | - | MTTLRDKKQ | - | MTTLRDKKQ | - | MTTLRDKKQ |
|  | - | - | - | NEKDLLELD | NEKDLLELD | NEKDLLELD |
|  | - | - | - | QEDVISLWD | QEDVISLWD | QEDVISLWD |
|  | - | - | - | SVRLVNGFL | SVRLVNGFL | SVRLVNGFL |
|  | - | SIVNRVRQG | SIVNRVRQG | - | SIVNRVRQG | - |
|  | - | - | - | RSSCKGLRR | RSSCKGLRR | RSSCKGLRR |
|  | - | - | - | RVVEREKRA | RVVEREKRA | RVVEREKRA |
|  | - | - | - | RYLKDQKFL | RYLKDQKFL | RYLKDQKFL |
|  | - | - | TLKQVIGKL | TLKQVIGKL | - | TLKQVIGKL |
|  | - | - | TPLCVTLNC | TPLCVTLNC | - | TPLCVTLNC |
|  | - | - | VIKQACPKI | VIKQACPKI | - | VIKQACPKI |
|  | - | - | - | VNRVRQGYS | VNRVRQGYS | VNRVRQGYS |
|  | - | - | - | VPTDPNPQE | VPTDPNPQE | VPTDPNPQE |
|  | - | - | VTVYYGVPV | VTVYYGVPV | - | VTVYYGVPV |
|  | - | - | - | WASLWNWFD | WASLWNWFD | WASLWNWFD |
|  | WATHACVPT | WATHACVPT | WATHACVPT | - | - | - |
|  | - | - | - | WEREISNYT | WEREISNYT | WEREISNYT |
|  | - | - | - | WFDITRWLW | WFDITRWLW | WFDITRWLW |
|  | WGCSGKIIC | WGCSGKIIC | WGCSGKIIC | - | - | - |
|  | - | - | - | WNNMTWTEW | WNNMTWTEW | WNNMTWTEW |
|  | - | - | - | WPNLWKWGT | WPNLWKWGT | WPNLWKWGT |
|  | - | WRSLYKYKV | WRSLYKYKV | WRSLYKYKV | - | - |
|  | - | - | - | YEEIWNNMT | YEEIWNNMT | YEEIWNNMT |
|  | - | - | - | YHRLRDLIL | YHRLRDLIL | YHRLRDLIL |
|  | - | - | - | QLLSGIVQQ | QLLSGIVQQ | QLLSGIVQQ |
|  |  | - | AAGRAILHI | - | AAGRAILHI | - |
|  | - | - | AARTVELLG | - | AARTVELLG | - |
|  | - | - | - | ACPKISFDP | - | ACPKISFDP |
|  | - | - | - | AGYAILKCN | - | AGYAILKCN |
|  | - | - | - | AIAITVAGW | - | AIAITVAGW |
|  | - | AIEAQQHLL | AIEAQQHLL | - | - | - |
|  | AIIIRSENL | AIIIRSENL | - | - | - | - |
|  | AQQHLLQLT | - | AQQHLLQLT | - | - | - |
|  | - | AQRAWRAIL | AQRAWRAIL | - | - | - |
|  | AKTIIVHLN | AKTIIVHLN | - | - | - | - |
|  | - | - | - | AISLFNAIA | - | AISLFNAIA |
|  | - | AWDDLRSLC | - | - | AWDDLRSLC | - |
|  | CLFSYHRLR | - | CLFSYHRLR | - | - | - |
|  | - | - | - | CSFNMTTEL | - | CSFNMTTEL |
|  | - | - | - | CTHGIKPVV | - | CTHGIKPVV |
|  | CVKLTPLCV | CVKLTPLCV | - | - | - | - |
|  | - |  | - | CTPAGYAIL | - | CTPAGYAIL |
|  | CVSNITGIL | CVSNITGIL | - | - | - | - |
|  | DGGANNTTN | - | DGGANNTTN | - | - | - |
|  | DIIGDIRKA | DIIGDIRKA | - | - | - | - |
|  | - |  | - | DKNFNGTGP | - | DKNFNGTGP |
|  | DNWRSELYK | - | DNWRSELYK | - | - | - |
|  | - | - | - | DRDRSVRLV | - | DRDRSVRLV |
|  | DRNEKDLLL | - | DRNEKDLLL | - | - | - |
|  | - | - | - | EDGGEQDRD | - | EDGGEQDRD |
|  | - | - | - | EEIWNNMTW | - | EEIWNNMTW |
|  | - | - | - | EIHLENVTE | - | EIHLENVTE |
|  | - | - | - | EIIGDIRKA | - | EIIGDIRKA |
|  | ELLGHSSLK | - | - | - | ELLGHSSLK | - |
|  | - | - | - | ELRDKKQKV | - | ELRDKKQKV |
|  | ENLTNNAKT | ENLTNNAKT | - | - | - | - |
|  | EPLGIAPTR | - | EPLGIAPTR | - | - | - |
|  | - | - | - | ERTEEDGGE | - | ERTEEDGGE |
|  | IFIMIVGGL | EVRNCSFNM | - | - | - | - |
|  | - | - | - | EYRLINCNT | - | EYRLINCNT |
|  | - | - | - | FDPIPIHYC | - | FDPIPIHYC |
|  | - | - | - | FYWGQELKI | - | FYWGQELKI |
|  | FGFLGAAGS | FGFLGAAGS | - | - | - | - |
|  | - | FLALAWDDL | FLALAWDDL | - | - | - |
|  | FQTPTHHQR | FQTPTHHQR | - | - | - | - |
|  | - | FILIAARTV | FILIAARTV | - | - | - |
|  | - | FYRTGDIIG | FYRTGDIIG | - | - | - |
|  | - | GAAGSTMGA | GAAGSTMGA | - | - | - |
|  | - | GQVFYRTGD | GQVFYRTGD | - | - | - |
|  | - | HSSLKGLRR | HSSLKGLRR | - | - | - |
|  | - | HLNKSVINC | HLNKSVINC | - | - | - |
|  | - | HNVWATHAC | HNVWATHAC | - | - | - |
|  | - | IFIMIVGGL | IFIMIVGGL | - | - | - |
|  | - | - | - | - | GEFFYCNTT | GEFFYCNTT |
|  | GGGEQGRDR | GGGEQGRDR | - | - | - | - |
|  | GFLALAWDD | - | GFLALAWDD | - | - | - |
|  | GGEQGRDRS | - | - | - | GGEQGRDRS | - |
|  | - | GGNNNNTNE | GGNNNNTNT | - | - | - |
|  | - | GIGAMIFGF | GIGAMIFGF | - | - | - |
|  | - | - | - | GKVFYRTGE | - | GKVFYRTGE |
|  | GLWGCSGKI | - | GLWGCSGKI | - | - | - |
|  | - | - | - | GNSSNNETF | - | GNSSNNETF |
|  | - | - | - | GQELKISAI | - | GQELKISAI |
|  | - | - | - | - | GSINCVSNI | GSINCVSNI |
|  | GTKWNETLK | - | - | GTKWNETLK | - | - |
|  | - | - | - | GVGQAMYAP | - | GVGQAMYAP |
|  | - | - | - | - | HFNCRGEFF | HFNCRGEFF |
|  | HYCTPAGYA | HYCTPAGYA | - | - | - | - |
|  | ICTTAVPWN | - | - | - | ICTTAVPWN | - |
|  | - | - | - | - | IENNNSSEY | IENNNSSEY |
|  | ASITLTVQA | - | ASITLTVQA | - | - | - |
|  | - | IICTTAVPW | IICTTAVPW | - | - | - |
|  | KDLLLDKWA | KDLLLDKWA | - | - | - | - |
|  | - | - | - | KVFYRTGEI | - | KVFYRTGEI |
|  | KYKVVQIEP | - | KYKVVQIEP | - | - | - |
|  | KVVQIEPLG | KVVQIEPLG | - | - | - | - |
|  | LRAIEAQQH | - | LRAIEAQQH | - | - | - |
|  | - | - | - | LWDQSLKPC | - | LWDQSLKPC |
|  | - | - | LRDKKKQAY | - | LRDKKKQAY | - |
|  | IGGGQGRDR | IGGGQGRDR | - | - | - | - |
|  | - | - | IINMWQGVG | - | IINMWQGVG | - |
|  | - | - | IIRSENLTN | - | IIRSENLTN | - |
|  | - | - | IKQACPKIS | - | IKQACPKIS | - |
|  | - | - | ILAVERYLK | - | ILAVERYLK | - |
|  | ILIAARTVL | ILIAARTVL | - | - | - | - |
|  | - | IVHLNKSVI | IVHLNKSVI | - | - | - |
|  | - | - | - | IVQIENNNS | - | IVQIENNNS |
|  | - | IVQIDNNSS | - | - | IVQIDNNSS | - |
|  | - | - | - | KEHFNKTII | - | KEHFNKTII |
|  | - | KLFNNTCIG | - | - | KLFDNETTG | - |
|  | - | KLKHFNKTI | KLKHFNKTI | - | - | - |
|  | - | KQACPKISF | KQACPKISF | - | - | - |
|  | - | KSFIWNNMT | KSYIWNNMT | - | - | - |
|  | - | - | - | LAEEEIIIR | - | LAEEEIIIR |
|  | - | - | - | LCLFSYHRL | - | LCLFSYHRL |
|  | - | LDKWASLWN | LDKWASLWN | - | - | - |
|  | - | - | - | LIGLRIIFA | - | LIGLRIIFA |
|  | - | - | - | LKISAISLF | - | LKISAISLF |
|  | - | LINCNTSVI | - | - | LINCNTSVI | - |
|  | - | - | - | - | LKTSAISLL | LKTSAISLL |
|  | - | LLDKWASLW | LLDKWASLW | - | - | - |
|  | - | LLLNGSLAI | LLLNGSLAI | - | - | - |
|  | - | - | - | LNGSLAEEE | - | LNGSLAEEE |
|  | - | - | LNCNDATFT | - | LNCNDATFT | - |
|  | - | LLYWGQELK | LLYWGQELK | - | - | - |
|  | - | - | - | LSGIVQQQS | - | LSGIVQQQS |
|  | - | - | - | LTESQNQQD | - | LTESQNQQD |
|  | - | LWVTVYYGV | LWVTVYYGV | - | - | - |
|  | - | - | - | LWKWGTLIL | - | LWKWGTLIL |
|  | - | - | - | NCNSTIILP | - | NCNSTIILP |
|  |  | - | LLGLWGCSG | - | LLGLWGCSG | - |
|  | - | - | LKPCVKLTP | - | LKPCVKLTP | - |
|  |  | - | - | ISLFNAIAI | - | ISLFNAIAI |
|  | RGWEGLKYL | RGWEGLKYL | - | - | - | - |
|  | RINCVSNIT | RINCVSNIT | - | - | - | - |
|  | - | - | - | NCNTSVIKQ | - | NCNTSVIKQ |
|  | - | NCRGFFYCN | NCRGFFYCN | - | - | - |
|  | - | NCTRPSNNT | NCTRPSNNT | - | - | - |
|  | - | - | - | NGFLALVWE | - | NGFLALVWE |
|  | - | NGSLAIIIR | NGSLAIIIR | - | - | - |
|  | - | NGTIILPCK | NGTIILPCK | - | - | - |
|  | - | NGTKWNTLK | NGTKWNTLK | - | - | - |
|  | - | - | - | NIIGNITDE | - | NIIGNITDE |
|  | - | NIKDNWRSL | NIKDNWRSL | - | - | - |
|  | - | NITGILLTR | NITGILLTR | - | - | - |
|  | - | - | - | NKTIIFQPP | - | NKTIIFQPP |
|  | - | NSSYRLINC | NSSYRLINC | - | - | - |
|  | - | - | PCVKLTPLC | PCVKLTPLC | - | - |
|  | - | PIGNNSSYR | PIGNNSSYR | - | - | - |
|  | - | PPSGGDLIT | PPSGGDLIT | - | - | - |
|  | - | PRIGGGEQG | PRIGGGEQG | - | - | - |
|  | - | - | - | PSGGDLEIT | - | PSGGDLEIT |
|  | - | - | - | QIENNNSSE | - | QIENNNSSE |
|  | - | - | - | QIYNILTES | - | QIYNILTES |
|  | - | - | - | QMNWPNLWK | - | QMNWPNLWK |
|  | - | RAWRAILHI | RAWRAILHI | - | - | - |
|  | - | RIRQGLRAL | RIRQGLRAL | - | - | - |
|  | - | - | - | RILAVERYL | - | RILAVERYL |
|  | - | - | - | RMTIGPGKV | - | RMTIGPGKV |
|  | - | RRVVRKRAV | RRVVRKRAV | - | - | - |
|  | - | RSLYKYKVV | RSLYKYKVV | - | - | - |
|  | - | - | - | - | RVRGTQMNW | RVRGTQMNW |
|  | - | SDAKAHTEV | SDAKAHTEV | - | - | - |
|  | - | - | - | SDNLWVTVY | - | SDNLWVTVY |
|  | - | - | - | SFNMTTELR | - | SFNMTTELR |
|  | - | - | - | SLKPCVKLT | - | SLKPCVKLT |
|  | - | - | - | SLKYLGNLL | - | SLKYLGNLL |
|  | - | SNLLRAIEA | - | - | SNLLRAIEA | - |
|  | - | SNYTNQIYI | SNYTNQIYI | - | - | - |
|  | - | - | - | SPLSFQIPT | - | SPLSFQIPT |
|  | - | - | - | SQLFNCTNC | - | SQLFNCTNC |
|  | - | - | - | SQLFNTSQL | - | SQLFNTSQL |
|  | - | - | - | SYEEIWNNM | - | SYEEIWNNM |
|  | - | - | - | - | SVKINCTRP | SVKINCTRP |
|  | - | - | - | STQLLLNGS | - | STQLLLNGS |
|  | - | - | TGPCNDTII | - | TGPCNDTII | - |
|  | - | - | - | TGPCKNVSS | - | TGPCKNVSS |
|  | - | - | TIGNITDVR | - | TIGNITDVR | - |
|  | - | TLRDKKQKV | TLRDKKQKV | - | - | - |
|  | - | TNANLTNSN | TNANLTNSN | - | - | - |
|  | - | - | - | TNCNSTIIL | - | TNCNSTIIL |
|  | - | - | - | TNIIGNITD | - | TNIIGNITD |
|  | - | - | - | TNVTNTNNT | - | TNVTNTNNT |
|  | - | TNWLWYIKI | TNWLWYIKI | - | - | - |
|  | - | - | - | TQLLLNGSL | - | TQLLLNGSL |
|  | - | - | TRPSNNTRT | - | TRPSNNTRT | - |
|  | - | - | - | VFYRTGEII | - | VFYRTGEII |
|  | - | - | - | VGIGAMIFG | - | VGIGAMIFG |
|  | - | VLLGHSSLK | VLLGHSSLK | - | - | - |
|  | - | - | VRKRAVGIG | - | VRKRAVGIG | - |
|  | - | VTENFNMWK | VTENFNMWK | - | - | - |
|  | - | - | - | WEDLRSLCL | - | WEDLRSLCL |
|  | - | - | - | WESLKYLGN | - | WESLKYLGN |
|  | - | - | WGQLKIGLV |  | WGQLKIGLV | - |
|  | - | - | - | WNETLKQVI | - | WNETLKQVI |
|  | - | WQGVGQAMY | WQGVGQAMY | - | - | - |
|  | - | - | - | - | WRWGTWILG | WRWGTWILG |
|  | - | YKLDIVQID | - | - | YKLDIVQID | - |
|  | - | YLKDQKFLG | YLKDQKFLG | - | - | - |
|  | - | YYGVPVWRD | YYGVPVWRD | - | - | - |
| **Novel epitopes** | - | - | - | - | AENLWVTVH | - |
|  | - | - | - | - | AFYRTGEIT | - |
|  | - | AGSTMGAAS | - | - | - | - |
|  | - | - | AKKLKHFNK | - | - | - |
|  | AGQAMYAPP | - | - | - | - | - |
|  | - | - | - | - | APAGFAILK | - |
|  | AMYAPPISG | - | - | - | - | - |
|  | ANNTTNETF | - | - | - | - | - |
|  | APPISGRIN | - | - | - | - | - |
|  | ARQLLSGIV |  |  |  |  |  |
|  | - | CIGNTMGCN | - | - | - | - |
|  | - | CNGTIILPC | - | - | - | - |
|  | - | - | - | - | CTNVSSVQC | - |
|  | - | - | CTRPSNNTR | - | - | - |
|  | - | - | DDLRSLCLF | - | - | - |
|  | CTTAVPWNS | - | - | - | - | - |
|  | - | - | - | - | DEIWKNMTW | - |
|  | - | - | - | - | DDYMLINCN | - |
|  | - | - | DFILIAART | - |  | - |
|  | - | - | DIRKAYCIN | - |  | - |
|  | - | - | - | - | DIVQIDEKK | - |
|  | - | - | - | - | DKKKQAYAL | - |
|  | - | DLITMHHFN | - | - | - | - |
|  | - | - | - | - | DLISIVARA | - |
|  | DIVQINNSS | - | - | - | - | - |
|  | - | - | DLRSLCLFS | - | - | - |
|  | - | - | DNNNTIGNI | - | - | - |
|  | DRPERIGGG | - | - | - | - | - |
|  | - | - | DQKFLGLWG | - | - | - |
|  | - | - | - | - | EDFNMWKNK | - |
|  |  | - | - | - | EEEIIIRSE | - |
|  | - | - | - | - | EFFYCNTTK | - |
|  | - | - | - | - | EGGGEQGRD | - |
|  | - | - | - | - | EGIEEGGGE | - |
|  | - | - | - | - | EKKKDDYML | - |
|  | - | - | - | - | EQHEDVISL | - |
|  | - | - | - | - | EQVKKKLGK | - |
|  | - | - | - | - | EREKRAVGL | - |
|  | - | ERISNYTNQ | - | - | - | - |
|  | - | - | - | - | EVTMHHFTC | - |
|  | EGLKYLGNL | - | - | - | - | - |
|  |  | EQGRDRSVR | - | - | - | - |
|  | EITMHHFNC | - | - | - | - | - |
|  | EKDLLLDKW | - | - | - | - | - |
|  | - | - | - | - | FYCNTTKLF | - |
|  | - | - | FNSTWIGNT | - | - | - |
|  | - | - | FDITNWLWY | - | - | - |
|  | - | - | FDPIPIHYC | - | - | - |
|  | FIWNNMTWI | - | - | - | - | - |
|  | - | - | - | FNAIAITVA | - | - |
|  | FYKLDIVQI | - | - | - | - | - |
|  | FNNTCIGNT | - | - | - | - | - |
|  | - | - | - | - | FTCRGEFFY | - |
|  | - | - | - | - | GRELKTSAI | - |
|  |  | IWERISNYT | - | - | - | - |
|  | - | - | - | IGPGKVFYR | - | - |
|  | - | - | - | - | IIWRAILHI | - |
|  | - | - | IKDNWRSLY | - | - | - |
|  | - | - | - | - | KLTNKTVIF | - |
|  | - | - | - | - | KTSAISLLD | - |
|  | - | - | - | KTIIVHLNK | - | - |
|  | - | - | - | - | KVLEQVKKK | - |
|  | - | - | KWNTLKQVA | - | - | - |
|  | - | LGHSSLKGL | - | - | - | - |
|  | - | - | LQARVLAVR | - | - | - |
|  | KVLKQVTKL | - | - | - | - | - |
|  | - | GCNGTIILP | - | - | - | - |
|  | - | - | - | - | GFLGAAGST | - |
|  | - | - | - | - | GFAILKCNN | - |
|  | - | - | - | GGGNIKDNW | - | - |
|  | - | - | - | GGQGRDRSV | - | - |
|  | - | - | GGTNGTIIL | - | - | - |
|  | - | - | - | - | GILLTRDGG | - |
|  | - | GHSSLKGLR | - | - | - | - |
|  | - | - | - | - | GIINCVSNI | - |
|  | GAGQAMYAP | - | - | - | - | - |
|  | GAMIFGFLG | - | - | - | - | - |
|  | GAWRAILHI | - | - | - | - | - |
|  | GDLEITMHH | - | - | - | - | - |
|  | GGDLEITMH | - | - | - | - | - |
|  | GKIICTTAV | - | - | - | - | - |
|  | - | - | GNITDVRNC | - | - | - |
|  | - | - | - | GNNSSYRLI | - | - |
|  | - | - | - | GNTTGGHNG | - | - |
|  | GFFYCNTTQ | - | - | - | - | - |
|  |  | - | - | - | GPCNDTIIL | - |
|  | GNTMGCNGT | - | - | - | - | - |
|  | - | - | - | GQGRDRSVR | - | - |
|  | - | - | - | GQLKISAIS | - | - |
|  | - | - | - | GSTMGAASI | - | - |
|  | - | - | - | GTGPCKNVS | - | - |
|  | - | - | - | - | GTGPCTNVS | - |
|  | GWEGLKYLG | - | - | - | - | - |
|  | - | - | - | GYAILKCND | - | - |
|  | - | - | - | GYSPLSFQT | - | - |
|  | - | - | - | - | HGIKPVVST | - |
|  | - | - | HIGPGQVFY | - | - | - |
|  | - | - | - | - | HLNESVKIN | - |
|  | - | - | - | - | HMLQLTVWG | - |
|  | - | - | IAQQHLLQL | - | - | - |
|  | - | - | - | - | ICSAAENLW | - |
|  | - | IEPLGIAPT | - | - | - | - |
|  | - |  | IEVAQRAWR | - | - | - |
|  | - | - | - | - | IFEQPPPGG | - |
|  | - | - | IFQPPSGGD | - | - | - |
|  | - | - | IGDIRKAYC | - | - | - |
|  | - | - | IINCVSNIT | - | - | - |
|  | - | - | INMWQGVGQ | - | - | - |
|  | - | - | ITDVRNCSF | - | - | - |
|  | - | IRKAYCING | - | - | - | - |
|  | - | - | - | - | ITGDIRKAY | - |
|  | - | - | - | - | GCSGKIICT | - |
|  | - | GDIIGDIRK | - | - | - | - |
|  | GTITLPCKI | - | - | - | - | - |
|  | - | IVAQRAWRA | - | - | - | - |
|  | - | - | - | - | IVARAVELL | - |
|  | - | - | IVFQPPSGG | - | - | - |
|  | HHQRPDRPE | - | - | - | - | - |
|  | - | IVGGLIGLR | - | - | - | - |
|  | - | - | IWNNMTWIW | - | - | - |
|  | - | - | IWRISNYTN | - | - | - |
|  | - | IYILTSQNQ | - | - | - | - |
|  | - | - | - | - | - | KISAISLFN |
|  | - | - | KGLRRGWGL | - | - |  |
|  | - | - | - | - | KKQAYALFY | - |
|  | - | - | - | - | KKKLGKLTN | - |
|  | - | - | - | - | KLLGLWGCS |  |
|  | - | - | - | - | - | KNNMVEQMQ |
|  | - | - | - | - | KNEKDLLEL | - |
|  | - | - | - | - | KNKMVEQHE | - |
|  | - | - | - | - | KQAYALFYK | - |
|  | IFGFLGAAG | - | - | - | - | - |
|  | IGAMIFGFL | - | - | - | - | - |
|  | IGLRIIFAV | - | - | - | - | - |
|  | IIGDIRKAY | - | - | - | - | - |
|  | IIIRSENLT | - | - | - | - | - |
|  | IKPVVSTQL | - | - | - | - | - |
|  | IKQLQARVL | - | - | - | - | - |
|  | IMIVGGLIG | - | - | - | - | - |
|  | - | - | LAIIIRSNL | - | - | - |
|  | - | LDIVQIDNN | - | - | - | - |
|  | - | - | - | - | LEVTMHHFT | - |
|  | - | - | - | - | LFDNETTGP | - |
|  | - | LIAARTVLL | - | - | - | - |
|  | - | - | LKGLRRGWG | - | - | - |
|  | - | - | - | - | LGSILSYWG | - |
|  | INCTRPSNN | - | - | - | - | - |
|  | INGTKWNKV | - | - | - | - | - |
|  | ISGRINCVS | - | - | - | - | - |
|  | ITGILLTRD | - | - | - | - | - |
|  | ITIGPGQVF | - | - | - | - | - |
|  | ITLPCKIKQ | - | - | - | - | - |
|  | ITLTVQARQ | - | - | - | - | - |
|  | ITMHHFNCR | - | - | - | - | - |
|  | IVQQQSNLL | - | - | - | - | - |
|  | KGLRRGWEG | - | - | - | - | - |
|  | KIFIMIVGG | - | - | - | - | - |
|  | KIICTTAVP | - | - | - | - | - |
|  | KIKQIINMW | - | - | - | - | - |
|  | KISFDPIPI | - | - | - | - | - |
|  | KISAISLLD | - | - | - | - | - |
|  | KKQKVHALF | - | - | - | - | - |
|  | KLDIVQINN | - | - | - | - | - |
|  | KQLQARVLA | - | - | - | - | - |
|  | KQVTKLKHF | - | - | - | - | - |
|  | - | - | - | - | LISIVARAV | - |
|  | - | LLRAIEAQQ | - | - | - | - |
|  | - | - | LLRAIAQQH | - | - | - |
|  | - | - | LLSGIVQQQ | - | - | - |
|  | - | LNKSVINCT | - | - | - | - |
|  | - | LLTRDGGNN | - | - | - | - |
|  | - | - | LWNWFDITN | - | - | - |
|  | - | - | LWYIKIFIM | - | - | - |
|  | - | MGCNGTIIL | - | - | - | - |
|  | - | - | - | - | MTWVQWERE | - |
|  | - | MWQGVGQAM | - | - | - | - |
|  | LALAWDDLR | - | - | - | - | - |
|  | LAVERYLKD | - | - | - | - | - |
|  | LAWDDLRSL | - | - | - | - | - |
|  | LCVTLNCTN | - | - | - | - | - |
|  | LKCNDKNFN | - | - | - | - | - |
|  | LKHFNNKTI | - | - | - | - | - |
|  | LKDQKFLGL | - | - | - | - | - |
|  | LKISAISLL | - | - | - | - | - |
|  | LKYLGNLLL | - | - | - | - | - |
|  | LSFQTPTHH | - | - | - | - | - |
|  | LYWGQLKIS | - | - | - | - | - |
|  | MGAASITLT | - | - | - | - | - |
|  | MGCNGTITL | - | - | - | - | - |
|  | MIFGFLGAA | - | - | - | - | - |
|  | MRVKTQMNW | - | - | - | - | - |
|  | MWKNNMVEQ | - | - | - | - | - |
|  | MWQGAGQAM | - | - | - | - | - |
|  | NCSFNMTTL | - | - | - | - | - |
|  | NANLTNVNN | - | - | - | - | - |
|  | NCVSNITGI | - | - | - | - | - |
|  | NKTIIFQPP | - | - | - | - | - |
|  | NLLLYWGQL | - | - | - | - | - |
|  | NTMGCNGTI | - | - | - | - | - |
|  | NTTNETFRP | - | - | - | - | - |
|  | NTTQLFNNT | - | - | - | - | - |
|  | NVLKQVTKL | - | - | - | - | - |
|  | NVSNIIGNI | - | - | - | - | - |
|  | PERIGGGEQ | - | - | - | - | - |
|  | - | - | - | - | PLCVTLNCN | - |
|  | - | - | - | - | PPGGDLEVT | - |
|  | PGGGNIKDN | - | - | - | - | - |
|  | - | - | PDRPRIGGG | - | - | - |
|  | - | - | - | - | PCTNVSSVQ | - |
|  | - | - | PSNNTRTSI | - | - | - |
|  | - | - | - | - | PTDPNPQEI | - |
|  | QRPDRPERI | - | - | - | - | - |
|  | QSNLLRAIE | - | - | - | - | - |
|  | - | QDRNKDLLL | - | - | - | - |
|  | - | - | - | - | QCTHGIKPV | - |
|  | QVTKLKHFN | - | - | - | - | - |
|  | - | - | - | - | RAVELLGRS | - |
|  | - | - | RDKKQKVHA | - | - | - |
|  | RDFILIAAR | - | - | - | - | - |
|  | - | - | RDRSVRLVS | - | - | - |
|  | - | - | - | - | RDLISIVAR | - |
|  | - | - | - | - | RGPDRPEGI | - |
|  | RGFFYCNTT | - | - | - | - | - |
|  | RIGGGEQGR | - | - | - | - | - |
|  | RKRAVGIGA | - | - | - | - | - |
|  | - | - | RISNYTNQI | - | - | - |
|  | - | - | RKAYCINGT | - | - | - |
|  | - | - | RNCSFNMTT | - | - | - |
|  | - | - | - | - | RMWQGVGQA | - |
|  | RSELYKYKV | - | - | - | - | - |
|  | RSENLTNNA | - | - | - | - | - |
|  | RSFIWNNMT | - | - | - | - | - |
|  | - | - | RSNLTNNAK | - | - | - |
|  | - | - | - | RVKETQMNW | - | - |
|  | - | - | RVVRKRAVG | - | - | - |
|  | SITIGPGQV | - | - | - | - | - |
|  | SLAIIIRSE | - | - | - | - | - |
|  | SLCLFSYHR | - | - | - | - | - |
|  | SLKGLRRGW | - | - | - | - | - |
|  | - | SFIWNNMTW | - | - | - | - |
|  | - | - | - | - | SGFLALAWD | - |
|  | - | - | SLAIIIRSN | - | - | - |
|  | SSLKGLRRG | - | - | - | - | - |
|  | SSYRLINCN | - | - | - | - | - |
|  | STWSNRSFI | - | - | - | - | - |
|  | - | - | SYIWNNMTW | - | - | - |
|  | - | - | SYRLINCNT | - | - | - |
|  | TGDIIGDIR | - | - | - | - | - |
|  | TGILLTRDG | - | - | - | - | - |
|  | - | TIIFQPPSG | - | - | - | - |
|  | - | TIIVHLNKS | - | - | - | - |
|  | - | TKLFNNTCI | - | - | - | - |
|  | - | - | - | - | TKTIIVHLN | - |
|  | TIIFQPPSG | - | - | - | - | - |
|  | TIIVHLNKS | - | - | - | - | - |
|  | TITLPCKIK | - | - | - | - | - |
|  | - | - | TMGAASITL | - | - | - |
|  | - | TMGCNGTII | - | - | - | - |
|  | TLPCKIKQI | - | - | - | - | - |
|  | TMGCNGTIT | - | - | - | - | - |
|  | TNVNNTTNV | - | - | - | - | - |
|  | TQLFNNTCI | - | - | - | - | - |
|  | TTLRDKKQK | - | - | - | - | - |
|  | TTNETFRPG | - | - | - | - | - |
|  | TTNVSNIIG | - | - | - | - | - |
|  | - | - | TNGTIILPC | - | - | - |
|  | - | - | TNNAKTIIV | - | - | - |
|  | - | - | TNNNTTDNN | - | - | - |
|  | - | TNSNNTNGS | - | - | - | - |
|  | TTQLFNNTC | - | - | - | - | - |
|  | TVELLGHSS | - | - | - | - | - |
|  | TVQARQLLS | - | - | - | - | - |
|  | TVWGIKQLQ | - | - | - | - | - |
|  | TWIWREISN | - | - | - | - | - |
|  | - | - | - | - | TPTIGNLTG | - |
|  | - | TPTHHQRPD | - | - | - | - |
|  | - | - | TQLFNSTWI | - | - | - |
|  | - | - | - | - | TTQTNSTPT | - |
|  | - | QLLLNGSLA | - | - | - | - |
|  | VLAVERYLK | - | - | - | - | - |
|  | VLSIVNRVR | - | - | - | - | - |
|  | VPWNSTWSN | - | - | - | - | - |
|  | VQIDNSSYR | - | - | - | - | - |
|  | VQIEPLGIA | - | - | - | - | - |
|  | VQQQSNLLR | - | - | - | - | - |
|  | WDDLRSLCL | - | - | - | - | - |
|  | - | - | - | - | VGLGAMIFG | - |
|  | - | - | VFQPPSGGD | - | - | - |
|  | WIWREISNY | - | - | - | - | - |
|  | WQGAGQAMY | - | - | - | - | - |
|  | WSNRSFIWN | - | - | - | - | - |
|  | YILTSQNQQ | - | - | - | - | - |
|  | - | VGQAMYAPP | - | - | - | - |
|  | - | - | VINCTRPSN | - | - | - |
|  | - | - | VKLTPLCVT | - | - | - |
|  | - | - | VPIGNNSSY | - | - | - |
|  | - | - | VRQGYSPLS | - | - | - |
|  | - | - | YCNTTQLFN | - | - | - |
|  | - | - | YRTGDIIGD | - | - | - |
